# Attentive Variational Information Bottleneck for TCR–peptide interaction prediction

**DOI:** 10.1093/bioinformatics/btac820

**Published:** 2022-12-26

**Authors:** Filippo Grazioli, Pierre Machart, Anja Mösch, Kai Li, Leonardo V Castorina, Nico Pfeifer, Martin Renqiang Min

**Affiliations:** Biomedical AI Group, NEC Laboratories Europe, Heidelberg 69115, Germany; Biomedical AI Group, NEC Laboratories Europe, Heidelberg 69115, Germany; Biomedical AI Group, NEC Laboratories Europe, Heidelberg 69115, Germany; Machine Learning Department, NEC Laboratories America, Princeton, NJ 08540, USA; School of Informatics, University of Edinburgh, Edinburgh EH8 9AB, UK; Methods in Medical Informatics, Department of Computer Science, University of Tübingen, Tübingen 72076, Germany; Machine Learning Department, NEC Laboratories America, Princeton, NJ 08540, USA

## Abstract

**Motivation:**

We present a multi-sequence generalization of Variational Information Bottleneck and call the resulting model Attentive Variational Information Bottleneck (AVIB). Our AVIB model leverages multi-head self-attention to implicitly approximate a posterior distribution over latent encodings conditioned on multiple input sequences. We apply AVIB to a fundamental immuno-oncology problem: predicting the interactions between T-cell receptors (TCRs) and peptides.

**Results:**

Experimental results on various datasets show that AVIB significantly outperforms state-of-the-art methods for TCR–peptide interaction prediction. Additionally, we show that the latent posterior distribution learned by AVIB is particularly effective for the unsupervised detection of out-of-distribution amino acid sequences.

**Availability and implementation:**

The code and the data used for this study are publicly available at: https://github.com/nec-research/vibtcr.

**Supplementary information:**

Supplementary data are available at *Bioinformatics* online.

## 1 Introduction

Predicting whether T cells recognize peptides presented on cells is a fundamental step towards the development of personalized treatments to enhance the immune response, like therapeutic cancer vaccines (Buhrman and Slansky, 2013; Corse *et al.*, 2011; Hundal *et al.*, 2020; McMahan *et al.*, 2006; Meng and Butterfield, 2002; Slansky *et al.*, 2000). In the human immune system, T cells monitor the health status of cells by identifying foreign peptides on their surface (Davis and Bjorkman, 1988; Krogsgaard and Davis, 2005). The T-cell receptors (TCRs) are able to bind to these peptides, especially if they originate from an infected or cancerous cell. The binding of TCRs—also known as TCR recognition—with peptides, presented by major histocompatibility complex (MHC) molecules in peptide-MHC (pMHC) complexes, constitutes a necessary step for immune response (Glanville *et al.*, 2017; Rowen *et al.*, 1996). Only if TCR recognition takes place can cytokines be released, which leads to the death of a target cell.

TCRs consist of an α- and a β-chain whose structures determine the interaction with the pMHC complex. Each chain consists of three loops, referred to as complementarity-determining regions (CDR1–3). It is believed that the CDR3 loops primarily interact with the peptide of a given pMHC complex (Feng *et al.*, 2007; La Gruta *et al.*, 2018; Rossjohn *et al.*, 2015). Supplementary Material S1 depicts the 3D structure of a TCR–pMHC complex.

Recent discoveries (Dash *et al.*, 2017; Lanzarotti *et al.*, 2019) have demonstrated that both the CDR3α- and β-chains carry information on the specificity of the TCR toward its cognate pMHC target. Obtaining information about paired TCR α- and β-chains requires specific and expensive experiments, like single-cell (SC) sequencing, which limits its availability. Conversely, the bulk sequencing of a population of cells reactive to a peptide is cheaper, but it only allows to gain information about either the α- or the β-chain.

In this work, we propose Attentive Variational Information Bottleneck (AVIB) to predict TCR–peptide interactions. AVIB is a multi-sequence generalization of Variational Information Bottleneck (VIB) (Alemi *et al.*, 2016). Notably, we introduce Attention of Experts (AoE), a novel method for combining single-sequence latent distributions into a joint multi-sequence latent encoding distribution using self-attention. Owing to its design, AoE can naturally leverage the abundant available data where either the CDR3α or the CDR3β sequence is missing when estimating the multi-sequence variational posterior. The model learns to predict whether the binding between the peptide and the TCR takes place or not.

Extensive experiments demonstrate that AVIB significantly outperforms state-of-the-art methods. In addition, the probabilistic nature of the VIB framework allows to provide estimates on the uncertainty of AVIB’s predictions. We empirically show that AVIB can be used for out-of-distribution (OOD) detection of amino acid sequences without supervision.

### 1.1 Background and related works

#### 1.1.1 TCR–pMHC and TCR–peptide interaction prediction

Several recent works have investigated TCR–pMHC and TCR–peptide interaction prediction. Various proposed approaches operate simple CDR3β sequence alignment (Chronister *et al.*, 2021; Wong *et al.*, 2019). TCRdist computes CDR similarity-weighted distances (Dash *et al.*, 2017). SETE adopts k-mer feature spaces in combination with principal component analysis and decision trees (Tong *et al.*, 2020). Various methods adopt Random Forest to operate classification (De Neuter *et al.*, 2018; Gielis *et al.*, 2019; Springer *et al.*, 2020). ImRex tackles the problem with a method based on convolutional neural networks (CNNs) (Moris *et al.*, 2021). TCRGP is a classification method which leverages a Gaussian process (Jokinen *et al.*, 2019). ERGO is a deep learning approach which adopts long short-term memory networks and autoencoders to compute representations of peptide and CDR3β (Springer *et al.*, 2020). ERGO II (Springer *et al.*, 2021) is an updated version of ERGO which considers additional input data, i.e. CDR3α sequence, V and J genes, MHC and T-cell type. NetTCR-1.0 (Jurtz *et al.*, 2018) and NetTCR-2.0 (Montemurro *et al.*, 2021) propose a simple 1D CNN-based model, integrating peptide and CDR3 sequence information for the prediction of TCR–peptide specificity. TITAN (Weber *et al.*, 2021) is a bimodal neural network that explicitly encodes β-chain and peptide; it leverages transfer learning and SMILES (Weininger *et al.*, 1989) encoding to achieve good generalization.

#### 1.1.2 Deep multimodal variational inference

The problem investigated in this work consists in predicting whether multiple sequences of amino acids, i.e. a peptide and the CDR3s, bind. A single sequence is not informative of whether binding takes place or not when observed alone. As a consequence, binding prediction cannot be framed as a classical multimodal learning problem. Nevertheless, this work presents a strong relationship with multimodal variational inference and takes inspiration from it. In this section, related works from both the supervised and self-supervised learning domains are presented.

**Self-supervised learning.** Deep neural networks proved to be successful at modeling probability distributions in the context of Variational Bayes (VB) methods. The Variational Autoencoder (VAE) (Kingma and Welling, 2013) jointly trains a generative model from latent variables to observations with an inference network from observations to latent variables. Multimodal generalizations of the VAE shall tackle the problem of learning a joint posterior distribution of the latent variable conditioned on multiple input modalities. The Multimodal Variational Autoencoder (MVAE) (Wu and Goodman, 2018) models the joint posterior as a Product of Experts (PoE) over the marginal posteriors, enabling cross-modal generation at test time. The Mixture-of-experts Multimodal Variational Autoencoder (MMVAE) (Shi *et al.*, 2019) factorizes the joint variational posterior as a combination of unimodal posteriors, using a Mixture of Experts (MoE). MoE-based models have been used in the biomedical field to tackle challenges such as protein–protein interactions (Qi *et al.*, 2007), biomolecular sequence annotation (Caragea *et al.*, 2009) and clustering cell phenotypes from SC data (Kopf *et al.*, 2021). Their main advantage is that they can infer global patterns in the genetic or peptide sequences in supervised and unsupervised settings (Kopf *et al.*, 2021).

**Supervised learning.** The VIB (Alemi *et al.*, 2016) is to supervised learning what the β-VAE (Higgins *et al.*, 2017) is to unsupervised learning. VIB leverages variational inference to construct a lower bound on the Information Bottleneck (IB) objective (Tishby *et al.*, 2000). By applying the reparameterization trick (Kingma and Welling, 2013), Monte Carlo sampling is used to get an unbiased estimate of the gradient of the VIB objective. This allows using stochastic gradient descent to optimize the objective. Various multimodal generalizations of the VIB have been recently proposed: the Multimodal Variational Information Bottleneck (MVIB) (Grazioli *et al.*, 2022a) and DeepIMV (Lee and Schaar, 2021). Both MVIB and DeepIMV adopt the PoE to estimate a joint multimodal latent encoding distribution from the unimodal latent encoding distributions. In contrast, our AVIB model predicts interactions among multiple input sequences. This involves modeling complex relations among different sequences (analogous to but not the same as modalities) with powerful and flexible multi-head self-attention, for which PoE is a sub-optimal choice.

## 2 Materials and methods

Let *Y* be a random variable representing a ground truth label associated with an input random variable *X*. Let *Z* be a stochastic encoding of *X* defined by an encoder $p_{\theta }(Z|x)$ parameterized by a neural network. (Notation: *X*, *Y*, *Z* are random variables; ***x***, ***y***, ***z*** are their realizations; f(·;θ) and $p_{\theta }(\cdot )$ are functions and probability distributions parameterized by a vector θ; S represents a set.) Following Tishby *et al.* (2000), our goal consists in learning an encoding *Z* which is (a) maximally informative about *Y* and (b) maximally compressive about *X*. Using an information theoretic approach, we obtain the IB objective with p(X,Y,Z)=p(Z|X)p(Y|X)p(X):

(1) $$\underset{\theta }{\max}\;R_{IB}(\theta )=I(Z,Y;\theta )-\beta I(Z,X;\theta )\;,$$

where β≥0 is a Lagrange multiplier controlling the trade-off between (a) and (b) and I(Z,Y;θ) is the mutual information between *Z* and *Y* parameterized by θ:

(2) $$I(Z,Y;\theta )=\int p(z,y|\theta )\;\text{log}\;\frac{p(z,y|\theta )}{p(z|\theta )p(y|\theta )}dy\;dz\;.$$

As derived in Alemi *et al.* (2016), assuming $q_{\phi }(y|z)$ and $r_{\omega }(z)$ are variational approximations of the true p(y|z) and p(z), respectively, Equation 1 can be rewritten as:

(3) $$\begin{array}{c} J_{\mathrm{VIB}}=\frac{1}{N}\sum_{n=1}^{N}E_{\epsilon ∼p(\epsilon )}[-\text{log}\;q_{\phi }(y^{n}|f(x^{n},\epsilon ;\theta ))]\\ +\beta D_{KL}\left(p_{\theta }(Z|x^{n})||r_{\omega }(Z)\right)\;, \end{array}$$

where ϵ∼N(0,I) is an auxiliary Gaussian noise variable, *D*_KL_ is the Kullback–Leibler divergence and f(·;θ) is a vector-valued parametric deterministic encoding function (here a neural network). The reparameterization trick (Kingma and Welling, 2013) introduces ϵ and allows writing $p_{\theta }(z|x)dz=p(\epsilon )d\epsilon$, where z=f(x,ϵ;θ) is now treated as a deterministic variable. This formulation allows the noise variable to be independent of the model parameters and to compute gradients of the objective in Equation 3 and optimize via backpropagation. In this work, we let the latent encoding distribution on *Z* be a multivariate Gaussian distribution with a diagonal covariance structure $z∼p_{\theta }(Z|x)=N(\mu ,\mathrm{diag}(\sigma^{2}))$; a valid reparameterization is z=μ+σ⊙ϵ. With the variational distribution $r_{\omega }(Z)$ set to a standard multivariate Gaussian distribution N(0,I) as done in practice, we can view VIB as a variational encoder–decoder analogous to VAE (Kingma and Welling, 2013), in which the latent encoding distribution $p_{\theta }$ can be viewed as a latent posterior, and the variational decoding distribution $q_{\phi }$ can be viewed as a decoder.

In the same spirit of extending VAE (Kingma and Welling, 2013) to MVAE (Wu and Goodman, 2018), the VIB objective of Equation 3 can be generalized by representing *X* as a collection of multiple input sequences $X=\{X_{i}|i^{th}\;\mathrm{sequence}\;is\;\mathrm{present}\}$. In light of this, in the language of a variational encoder-decoder, the posterior $p_{\theta }(Z|x)$ of Equation 3 consists actually in the joint posterior $p_{\Theta }(Z|x_{1},\ldots ,x_{M}):=p_{\Theta }(Z|x_{1:M})$, conditioned on the joint *M* available sequences. However, for predicting the interaction label *Y* from *X*, the *M* different sequences cannot be simply treated as *M* different modalities.

### 2.1 Attention of experts

Similar to previous works (Grazioli *et al.*, 2022a; Wu and Goodman, 2018), the single-sequence posteriors are modeled as Gaussian distributions with diagonal structure: ${\tilde{q}}_{\theta_{i}}(Z|x_{i})=N(\mu_{i},\mathrm{diag}(\sigma_{i}^{2}))$. By stacking the parameters (represented as column-vectors) $\mu_{0}$ and $\sigma_{0}$ of the latent prior with the $\mu_{i}$ and $\sigma_{i}$ for all available i=1,…,M sequences, we define the following two matrices $M\in R^{(M+1)\times d_{Z}}$ and $\Sigma \in R^{(M+1)\times d_{Z}}$, where *d_Z_* is the dimensionality of the latent single-sequence posteriors:

(4) $$M=\left(\begin{array}{c} \mu_{0}^{T}\\ \vdots \\ \mu_{M}^{T} \end{array}\right)\;\;\;,\;\;\;\Sigma =\left(\begin{array}{c} \sigma_{0}^{T}\\ \vdots \\ \sigma_{M}^{T} \end{array}\right)\;\;.$$

We propose to implicitly learn the dependencies between the *M* single-sequence posteriors and the multi-sequence joint posterior by means of multi-head self-attention, leveraging its power of capturing multiple complex interactions in *X*, and allowing possible missing sequences:

(5) $$\{\begin{array}{c} \mu_{\mathrm{AoE}}=\mathrm{Pool}(\mathrm{MultiHea}d_{\mu }(M,M,M))\\ \sigma_{\mathrm{AoE}}=\mathrm{Pool}(\mathrm{MultiHea}d_{\sigma }(\Sigma ,\Sigma ,\Sigma )) \end{array}.$$

**Fig. 1.**
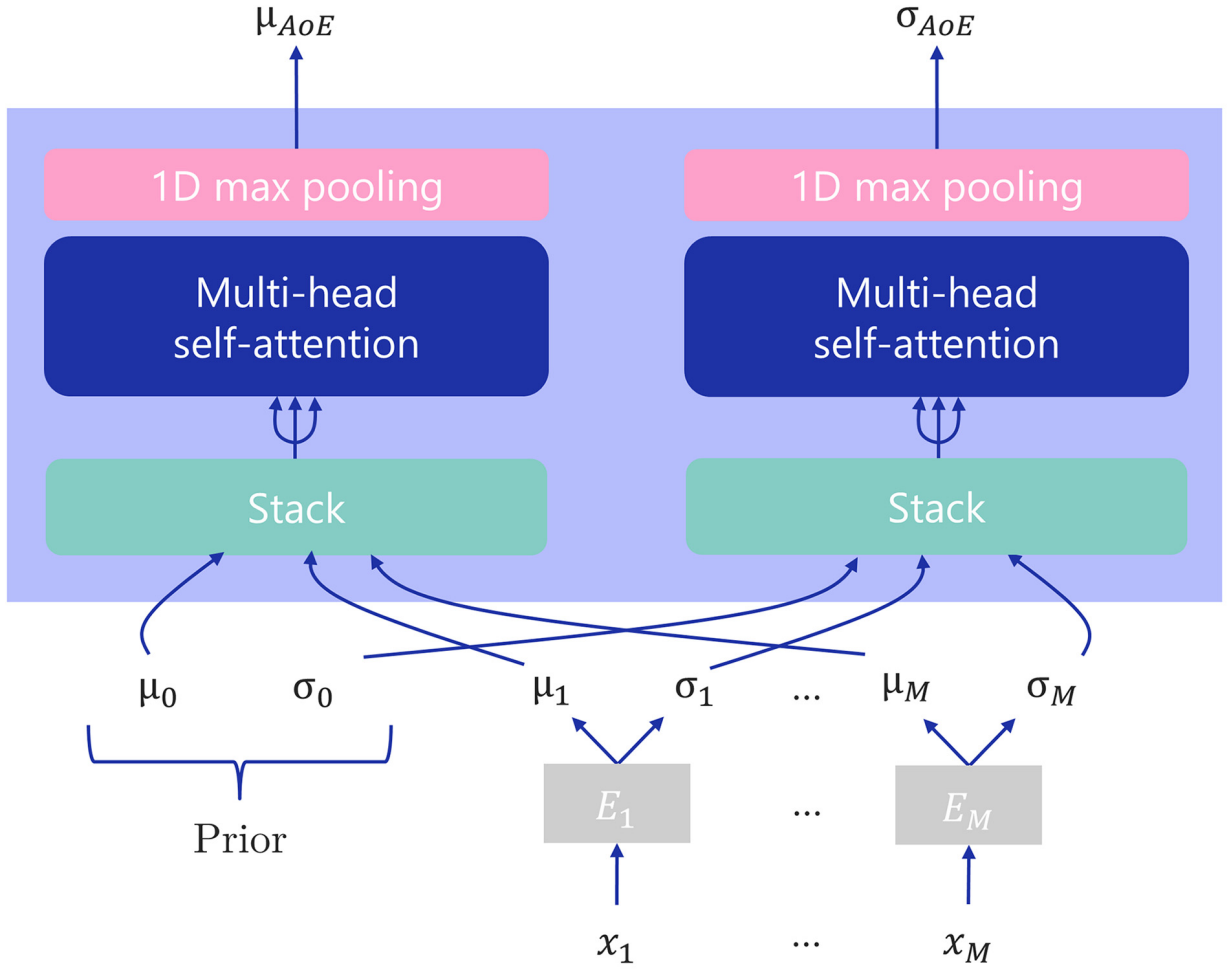
Attention of experts (AoE) for Gaussian posteriors. *E_i_* is the stochastic Gaussian encoder of the *i*th sequence

$\mathrm{Pool}:R^{(M+1)\times d_{Z}}\to R^{d_{Z}}$ is a 1D max pooling function. *MultiHead* is the standard multi-head attention block defined in Vaswani *et al.* (2017), whose equations are provided in Supplementary Material S2. We refer to Equation 5 as AoE. Figure 1 provides a schematic depiction of AoE.

We refer to a multi-sequence VIB which adopts AoE for modeling the multi-sequence joint posterior as AVIB. The AVIB objective is:

(6) $$\begin{array}{c} J_{\mathrm{AVIB}}=\frac{1}{N}\sum_{n=1}^{N}E_{\epsilon ∼p(\epsilon )}[-\text{log}\;q_{\phi }(y^{n}|f(x_{1:M}^{n},\epsilon ;\Theta ))]\\ +\beta D_{KL}\left(p_{\Theta }(Z|x_{1:M}^{n})||r_{\omega }(Z)\right))\;, \end{array}$$

where the multi-sequence posterior is modeled as $p_{\Theta }(Z|x_{1:M})=N(\mu_{\mathrm{AoE}},\mathrm{diag}(\sigma_{\mathrm{AoE}}^{2}))$.

Due to space limitation, we provide detailed description of the implementation, the training setup and the choice of the hyperparameter *β* in Supplementary Material S3. Supplementary Material S4 describes the full AVIB architecture.

### 2.2 Relation to multimodal variational inference

MVAE (Wu and Goodman, 2018) and MVIB (Grazioli *et al.*, 2022a) approximate the joint posterior $p_{\Theta }(Z|x_{1:M})$ assuming that the *M* modalities are conditionally independent, given the common latent variable *Z*. This allows expressing the joint posterior as a product of unimodal approximate posteriors ${\tilde{q}}_{\theta_{i}}(Z|x_{i})$ and a prior *p*(*Z*), referred to as PoE: $p_{\Theta }(Z|x_{1:M})=p(Z)\prod_{i=1}^{M}{\tilde{q}}_{\theta_{i}}(Z|x_{i})$, where $\Theta ={\left\{\theta_{i}\right\}}_{i=1}^{M}$. MMVAE (Shi *et al.*, 2019) factorizes the joint multimodal posterior as a mixture of Gaussian unimodal posteriors, referred to as MoE: $p_{\Theta }(Z|x_{1:M})=\frac{1}{M}\sum_{i=1}^{M}{\tilde{q}}_{\theta_{i}}(Z|x_{i})$.

PoE assumes conditional independence between modalities (Hinton, 2002). Furthermore, conditional dependence is impossible to capture by MoE, due to its additive form. This becomes a major shortcoming when modeling TCR–peptide interaction, in which the single sequences are not predictive of the binding if *observed individually*. Although AoE does not explicitly parameterize conditional dependence between the sequences, it does not assume that each sequence should be individually predictive of the class label, making it a more suitable candidate to model molecular interactions.

AoE can improve on PoE and MoE on multiple levels. First, employing attention for estimating the joint multi-sequence posterior allows learning relative importance among the various single-sequence posteriors. This allows dynamically enhancing the weight given to certain input sequences, while diminishing the focus on others, without being restrained to ‘AND’ and ‘OR’ relations, like PoE and MoE, respectively (Shi *et al.*, 2019). Second, as AoE is a parametric trainable module, it can learn to accommodate miscalibrated single-sequence posteriors, which are especially difficult to handle by PoE (Kutuzova *et al.*, 2021).

The adoption of PoE and MoE for the approximation of a multimodal posterior using unimodal encoders allows for inference also in case certain modalities are missing (Grazioli *et al.*, 2022a; Kutuzova *et al.*, 2021; Shi *et al.*, 2019; Wu and Goodman, 2018). A single encoder applied on the concatenation of all modalities would not allow that. Just like PoE and MoE, AoE allows inference with missing inputs. There is in fact no restriction on the number of rows of ***M*** and Σ (see Equations 4 and 5), which is the equivalent of the number of word tokens in a natural language processing setting (see Section 3.5).

In this work, we only benchmark AoE against PoE and do not compare against MoE. We believe MoE’s ‘OR’-nature is not suitable for modeling the chemical specificity of multiple molecules. If taken alone, the single-sequence variational posteriors are not informative of the chemical reaction. Analogously, sampling from a MoE—which has similarities to the OR operator—is not suitable for capturing how molecules chemically interact.

### 2.3 Information Bottleneck Mahalanobis distance

Although AVIB is not explicitly designed for uncertainty estimation, we propose a simple, yet effective, approach for OOD detection. This approach is strongly inspired by Lee *et al.* (2018) and leverages the Mahalanobis distance. In the following, we first summarize the method proposed by Lee *et al.* (2018). Then, we describe how to extend this approach to AVIB.

**Mahalanobis distance.** The Mahalanobis distance has proved to be an effective metric for OOD detection (Lee *et al.*, 2018). Let $f_{l}(x)$ denote the output of the *l*th hidden layer of a neural network, given an input ***x***. Using the training samples, this method fits a class-conditional Gaussian distribution to the embeddings of each class, computing a per-class mean $\mu_{l}^{c}=\frac{1}{N_{c}}\sum_{i:y_{i}=c}f_{l}(x_{i})$ and a shared covariance matrix $\Sigma_{l}=\frac{1}{N}\sum_{c=1}^{K}\sum_{i:y_{i}=c}(f_{l}(x_{i})-\mu_{l}^{c}){\left(f_{l}(x_{i})-\mu_{l}^{c}\right)}^{T}$. Given a test sample ***x***, the Mahalanobis score is computed as $\mathrm{scor}e_{\mathrm{Maha}}(x)=\sum_{l}\alpha_{l}M_{l}(x)$, where $M_{l}(x)=-\min_{c}\left(\frac{1}{2}(f_{l}(x)-\mu_{l}^{c})\Sigma_{l}^{-1}{\left(f_{l}(x)-\mu_{l}^{c}\right)}^{T}\right)$. Lee *et al.* (2018) fit the *α_l_* coefficients by training a logistic regression on a set of samples for which the knowledge of the OOD/ID label is assumed. Additionally, the authors show that adding a small (*ε*) controlled noise to the input can improve results, analogously to ODIN (Liang *et al.*, 2017).

We leverage the expectation of the learned latent posterior conditioned on all input sequences and fit *K* class-conditional Gaussian distributions using the ID training samples, where *K* is the number of classes. For the TCR–peptide interaction prediction task, we have two classes: binders and non-binders. The *K* empirical class means are computed as:

(7) $$\mu_{\mathrm{AVIB}}^{c}=\frac{1}{N_{c}}\sum_{i:y_{i}=c}E[Z|x_{1:M}^{i}]\;\;.$$

A shared covariance matrix is computed as:

(8) $$\begin{array}{c} \Sigma_{\mathrm{AVIB}}=\\ \frac{1}{N}\sum_{c=1}^{K}\sum_{i:y_{i}=c}(E[Z|x_{1:M}^{i}]-\mu_{\mathrm{AVIB}}^{c}){\left(E[Z|x_{1:M}^{i}]-\mu_{\mathrm{AVIB}}^{c}\right)}^{T}. \end{array}$$

Lee *et al.* (2018) compute Mahalanobis distances at multiple hidden layers of a neural network. A logistic regression is trained to learn relative weights assuming the knowledge of the ID/OOD label for a set of validation samples. In contrast to that, given a multi-sequence sample $x_{1:M}$, we propose to leverage the multi-sequence posterior distribution over encodings learned by AVIB, i.e. $p_{\Theta }(Z|x_{1:M})$ and compute one single Mahalanobis distance, which acts as OOD score:

(9) $$\begin{array}{c} \mathrm{scor}e_{\mathrm{AVIB}-\mathrm{Maha}}(x_{1:M})=\\ -\underset{c}{\min}(\frac{1}{2}(E[Z|x_{1:M}]-\mu_{\mathrm{AVIB}}^{c})\Sigma_{\mathrm{AVIB}}^{-1}{\left(E[Z|x_{1:M}]-\mu_{\mathrm{AVIB}}^{c}\right)}^{T}). \end{array}$$

This approach is hyperparameter free. Hence, it does not require a validation set for tuning. As a consequence, prior knowledge of OOD validation samples is not required.

## 3 Results and discussion

First, we provide a description of the datasets used in this work. We then apply AVIB to the TCR–peptide interaction prediction problem. Last, we demonstrate AVIB’s effectiveness in the context of OOD detection. All experiments are implemented using PyTorch (Paszke *et al.*, 2019). Code and data are publicly available at: https://github.com/nec-research/vibtcr.

### 3.1 Datasets

Recent studies (De Neuter *et al.*, 2018; Fischer *et al.*, 2020; Gielis *et al.*, 2019; Jokinen *et al.*, 2019; Jurtz *et al.*, 2018; Montemurro *et al.*, 2021; Moris *et al.*, 2021; Springer *et al.*, 2020; 2021; Tong *et al.*, 2020; Weber *et al.*, 2021; Wong *et al.*, 2019) investigate the prediction of TCR–peptide/–pMHC interactions. Most use data from the Immune Epitope Database (IEDB) (Vita *et al.*, 2019), VDJdb (Bagaev *et al.*, 2020) and McPAS-TCR (Tickotsky *et al.*, 2017), which mainly contain CDR3β data and lack information on CDR3α. We merge human TCR–peptide data extracted from the ERGO II and NetTCR-2.0 repositories (https://github.com/IdoSpringer/ERGO-II; https://github.com/mnielLab/NetTCR-2.0). Binding (i.e. positive) samples are derived from the IEDB, VDJdb and McPAS-TCR databases. Positive data points generated by Klinger *et al.* (2015), referred to as *MIRA set*, are also considered. (The MIRA set is publicly available in the NetTCR-2.0 repository https://github.com/mnielLab/NetTCR-2.0/tree/main/data.) We employ all non-binding (i.e. negative) samples used by Springer *et al.* (2021) and Montemurro *et al.* (2021). Hence, negative samples are derived from random recombination of positive data points, as well as from the 10× Genomics assays described in Montemurro *et al.* (2021). Overall, 271 366 human TCR–peptide samples are available. We organize the data and create the following datasets.

**α + β set.** 117 753 samples out of 271 366 present peptide information, along with both CDR3α and CDR3β sequences. In this work, we refer to this subset as the α+β*set*. The ground truth label is a binary variable which represents whether the peptide and TCR chains interact.

**β set.** 153 613 samples out of 271 366 present peptide and CDR3β information (the CDR3α sequence is missing). We refer to this subset as the *β set*. The *β set* and the α+β*set* are disjoint.

**Human TCR set.** We refer to the totality of the human TCR–peptide data (i.e. *β set* ∪ α+β*set*) as *Human TCR set*.

**Non-human TCR set.** We extract 5036 non-human TCR samples from the VDJdb database, which we use as OOD samples. These samples come from mice and macaques and present peptide and CDR3β information. We refer to these samples as *Non-human TCR set*.

In addition to the TCR datasets, in order to thoroughly evaluate AVIB on multiple types of molecular data, we perform experiments on the following peptide-MHC datasets.

**NetMHCIIpan-4.0 set.** This dataset consists of 108 959 peptide-MHC pairs and was proposed in Reynisson *et al.* (2020) for the training of the NetMHCIIpan-4.0 model. All MHC molecules are class II. A continuous binding affinity (BA) value, ranging in [0,1], is associated to each (peptide, MHC) pair and used to validate AVIB on a regression task.

**Human MHC set.** We create a second set of OOD samples composed of 463 684 peptide-MHC pairs. The peptide sequences are taken from the *Human TCR set*, i.e. the peptide information is shared among ID and OOD sets. The MHC molecules are represented as pseudo-sequences of amino acids. [For the MHC pseudo-sequences, we refer to the PUFFIN (Zeng and Gifford, 2019) repository: https://github.com/gifford-lab/PUFFIN/blob/master/data/pseudosequence.2016.all.X.dat.] We consider both Classes I and II MHC alleles. We refer to these samples as *Human MHC set*.

Supplementary Figure S7 depicts the distributions of the human TCR data for both the α+β*set* and the *β set*. The two datasets have similar peptide distributions but contain different CDR3β sequences. Supplementary Material S5.1 provides information regarding the distribution of the length of the amino acid sequences in the various datasets. Supplementary Material S5.2 provides information on the class distribution of the α+β*set* and *β set*. Supplementary Material S5.3 describes the binding affinity distributions of the *NetMHCIIpan-4.0 set*.

### 3.2 Pre-processing

In this work, peptides, CDR3α and CDR3β are represented as sequences of amino acids. The 20 amino acids translated by the genetic code are in general represented as English alphabet letters. Analogously to Montemurro *et al.* (2021), we pre-process the amino acid sequences using BLOSUM50 encodings (Henikoff and Henikoff, 1992), i.e. the substitution value of each amino acid represented by the BLOSUM50 matrix’ diagonal. This allows us to represent a sequence of *N* amino acids as a 20×N matrix, analogously to the approach proposed by Nielsen *et al.* (2003). After performing BLOSUM50 encoding, we standardize the features by subtracting the mean and scaling to unit variance. As the length of the amino acid sequences is not constant, we operate 0-padding after the BLOSUM50 encoding (Mösch and Frishman, 2021). This ensures that all matrices have shape $20\times N_{\max}$, where *N*_max_ is the length of the longest sequence. Information on the length distribution of the amino acid sequences can be found in Supplementary Material S5.1.

### 3.3 TCR–peptide interaction prediction

In order to evaluate AVIB’s performance on the TCR–peptide interaction prediction task, we perform experiments on three datasets: the α+β*set*, the *β set* and their union *β set* ∪ α+β*set*. For the *β set* and the union set, input samples are $\left(x_{\mathrm{Peptide}},x_{\mathrm{CDR}3\beta }\right)$ pairs. For the α+β*set*, inputs can be either $\left(x_{\mathrm{Peptide}},x_{\mathrm{CDR}3\beta }\right)$ pairs or $\left(x_{\mathrm{Peptide}},x_{\mathrm{CDR}3\alpha },x_{\mathrm{CDR}3\beta }\right)$ triples. For all tri-sequence experiments, we adopt a full multi-sequence extension of the *J*_AVIB_ objective (see Supplementary Material S6, Equation 12).

**Baselines.** We benchmark AVIB against two state-of-the-art deep learning methods for TCR–peptide interaction predictions: ERGO II (Springer *et al.*, 2021) and NetTCR-2.0 (Montemurro *et al.*, 2021). Additionally, we benchmark AVIB against the LUPI-SVM (Abbasi *et al.*, 2018), by leveraging the α-chain at training time as privileged information. For all benchmark methods, we adopt the original publicly available implementations (https://github.com/IdoSpringer/ERGO-II; https://github.com/mnielLab/NetTCR-2.0; https://github.com/wajidarshad/LUPI-SVM).

**Evaluation metrics.** Table 1 summarizes the experimental results. For evaluation, the area under the receiver operator characteristic (AUROC) curve, the area under the precision–recall (AUPR) curve and the F1 score (F1) are computed on the test sets. Five repeated experiments with different 80/20 training/test random splits are performed for robust evaluation.

**Table 1.** TCR–peptide interaction prediction results

Dataset	Inputs	Method	AUROC	AUPR	F1
*β set*	Pep + β	NetTCR-2.0	0.755 ± 0.001	0.395 ± 0.002	0.349 ± 0.002
		ERGO II	0.761 ± 0.011	0.415 ± 0.020	0.412 ± 0.010
		AVIB (ours)	**0.804 ± 0.001**	**0.494 ± 0.001**	**0.477 ± 0.001**
α+β *set*	Pep + β	LUPI-SVM	0.770 ± 0.001	0.212 ± 0.001	0.218 ± 0.001
		NetTCR-2.0	0.846 ± 0.002	0.396 ± 0.003	0.413 ± 0.001
		ERGO II	0.894 ± 0.001	**0.538 ± 0.004**	0.498 ± 0.003
		AVIB (ours)	**0.895 ± 0.001**	0.534 ± 0.004	**0.515 ± 0.002**
	Pep + α + β	NetTCR-2.0	0.862 ± 0.002	0.477 ± 0.003	0.472 ± 0.002
		ERGO II	0.903 ± 0.002	0.578 ± 0.004	0.528 ± 0.002
		AVIB (ours)	**0.913 ± 0.001**	**0.614 ± 0.002**	**0.586 ± 0.001**
*β set* ∪ α+β*set*	Pep + β	NetTCR-2.0	0.727 ± 0.001	0.342 ± 0.001	0.276 ± 0.002
		ERGO II	0.748 ± 0.015	0.379 ± 0.022	0.381 ± 0.014
		AVIB (ours)	**0.773 ± 0.001**	**0.414 ± 0.002**	**0.396 ± 0.003**

*Note*: The reported confidence intervals are standard errors over five repeated experiments with different independent training/test random splits. Reported scores are computed on the test sets. Baselines: NetTCR-2.0 (Montemurro *et al.*, 2021), ERGO II (Springer *et al.*, 2021) and LUPI-SVM (Abbasi *et al.*, 2018). Best results are in bold.

**Peptide+CDR3β.** On the *β set*, AVIB obtains ∼4% higher AUROC and 8% higher AUPR compared to the best baseline, ERGO II. On the *β set* ∪ α+β*set*, AVIB outperforms ERGO II by achieving ∼3% higher AUROC and ∼4% higher AUPR. On the α+β*set*, in the peptide+CDR3β setting, AVIB compares with ERGO II.

**Peptide+CDR3α.** Peptide+CDR3α results on the α+β*set* are reported in Supplementary Material S7.

**Peptide+CDR3α+CDR3β.** In the tri-sequence setting, when considering peptide and both CDR3α and CDR3β sequences, AVIB obtains 1% higher AUROC, ∼4% higher AUROC and ∼6% higher F1 score compared to the best baseline, ERGO II.

These experimental results demonstrate that AVIB is a competitive method for TCR–peptide interaction prediction. On the α+β*set*, AVIB’s tri-sequence (peptide+CDR3α+CDR3β) results outperform the results obtained in both bi-sequence (peptide+CDR3α and peptide+CDR3β) settings (see Table 1 and Supplementary Material S7). This shows that AVIB is an effective multi-sequence learning method, which can learn richer representations from the joint analysis of multiple data sequences.

#### 3.3.1 Cross-dataset experiments

In Supplementary Material S8, we present cross-dataset experiments, in which we train AVIB and the baseline models on the α+β*set* and test on the the *β set*. As shown in Supplementary Figure S7, the α+β*set* and the *β set* present similar peptide distributions, but contain different CDR3β sequences. Our cross-dataset results show that all models fail to generalize to unseen CDR3β sequences. These results are in line with Grazioli *et al.* (2022b), which analogously shows that state-of-the-art models fail to generalize to unseen peptides.

#### 3.3.2 Visualization of the attention weights

One of the advantages of using AoE for estimating the multi-sequence posterior is the dynamic weighting of the multiple single-sequence posteriors. This allows to capture relationships between the input sequences. In Supplementary Material S9, we show how the attention weights derived from the $\mu_{\mathrm{AoE}}$ self-attention block change while gradually mutating the peptide sequence. We notice, that as the peptide sequence disruption increases, the peptide-CDR3β attention weight drops while CDR3β-peptide increases.

### 3.4 Multi-sequence posterior approximation

In this section, we compare various techniques to approximate Gaussian joint posteriors. We perform experiments and benchmark on two datasets: α+β*set* and *NetMHCIIpan-4.0 set*. Experiments on the α+β*set* employ either $\left(x_{\mathrm{Peptide}},x_{\mathrm{CDR}3\beta }\right)$ pairs or $\left(x_{\mathrm{Peptide}},x_{\mathrm{CDR}3\alpha },x_{\mathrm{CDR}3\beta }\right)$ triples as inputs. Experiments on the *NetMHCIIpan-4.0 set* input $\left(x_{\mathrm{Peptide}},x_{\mathrm{MHC}}\right)$ pairs.

The ground truth labels of the *NetMHCIIpan-4.0 set* are continuous BA scores. For BA regression, we train models by substituting the log-likelihood of Equation 6 with a mean squared error (MSE) loss. BA prediction of pMHC complexes is—just like TCR–peptide interaction prediction—a fundamental problem in computational immuno-oncology (Cheng *et al.*, 2021; O’Donnell *et al.*, 2018, 2020; Reynisson *et al.*, 2020) and is a key step in the development of vaccines against cancer (Buhrman and Slansky, 2013; Corse *et al.*, 2011; Hundal *et al.*, 2020; McMahan *et al.*, 2006; Meng and Butterfield, 2002; Slansky *et al.*, 2000) and infectious diseases (Malone *et al.*, 2020). Peptides can only be presented on the surface of cells if they bind to MHC molecules. This mechanism allows the immune system to gain knowledge about in-cell anomalies such as cancerous mutations or viral infections.

**Baseline and ablation methods.** We benchmark AVIB, which employs AoE, against MVIB (Grazioli *et al.*, 2022a), which employs PoE. Additionally, we perform an ablation study meant to investigate the influence of multi-head self-attention in AoE. For the ablation, we remove the multi-head self-attention module from AoE (see Equation 5) and only operate a simple pooling of the various single-sequence posteriors. We define two ablation methods: Max Pooling of Experts (MaxPOOLoE), which adopts a 1D max pooling function and Average Pooling of Experts (AvgPOOLoE), which adopts 1D average pooling.

**Evaluation metrics.** For the evaluation of classification results on the α+β*set*, we adopt AUROC, AUPR, F1 and accuracy. For evaluating regression on the *NetMHCIIpan-4.0 set*, we employ MSE, root mean squared error (RMSE) and the *R*^2^ coefficient (Wright, 1921).

Table 2 presents classification and regression results on the α+β*set* and the *NetMHCIIpan-4.0 set*. AoE achieves best results in all settings and on both datasets. Interestingly, the ablation methods AvgPOOLoE and MaxPOOLoE (Supplementary Material S10) achieve worse performance compared to PoE.

**Table 2.** Multi-sequence posterior approximation—benchmark and ablation

Inputs	Metric	↑/↓	MVIB (PoE)	AvgPOOLoE	AVIB (AoE)
Pep + β	AUROC	↑	0.889 ± 0.001	0.883 ± 0.002	**0.895 ± 0.001**
	AUPR		0.512 ± 0.003	0.502 ± 0.002	**0.535 ± 0.004**
	F1		0.498 ± 0.002	0.484 ± 0.003	**0.515 ± 0.002**
	Accuracy		0.860 ± 0.003	0.852 ± 0.002	**0.873 ± 0.001**
Pep + α + β	AUROC	↑	0.910 ± 0.001	0.905 ± 0.002	**0.913 ± 0.001**
	AUPR		0.595 ± 0.004	0.589 ± 0.002	**0.614 ± 0.002**
	F1		0.575 ± 0.002	0.555 ± 0.007	**0.587 ± 0.001**
	Accuracy		0.907 ± 0.001	0.898 ± 0.002	**0.916 ± 0.001**
Pep + MHC II	MSE	↓	0.0313 ± 0.0001	0.0329 ± 0.0002	**0.0299 ± 0.0001**
	RMSE		0.137 ± 0.001	0.140 ± 0.001	**0.133 ± 0.003**
	*R* ^2^	↑	0.538 ± 0.001	0.514 ± 0.004	**0.559 ± 0.001**

*Note*: TCR–peptide binding prediction experiments are performed on the α+β*set*. Peptide-MHC BA regression experiments are performed on the *NetMHCIIpan-4.0 set*. Confidence intervals are standard errors over five repeated experiments with different training/test random splits. Best results are in bold. ↑: larger value is better. ↓: lower value is better.

PEP, peptide; α, CDR3α sequence; β, CDR3β sequence; MHC II, MHC Class II pseudo-sequence. Baseline, MVIB. Ablation without multi-head self-attention. AvgPOOLoE, Average Pooling of Experts.

### 3.5 Missing input sequences

In this section, we study AVIB’s performance when certain data sequences are available at training time, but missing at test time. We train AVIB on $\left(x_{\mathrm{Peptide}},x_{\mathrm{CDR}3\alpha },x_{\mathrm{CDR}3\beta }\right)$ triples from the α+β*set*. At test time, we omit one of the two CDR3 sequences. In real-world settings, it is in fact common to have batches of data where only CDR3α or CDR3β information is available. It is therefore efficient to leverage one single model which can operate also if a CDR3 sequence is missing. This prevents the need of training different models on the various sequences subsets.

**Fig. 2.**
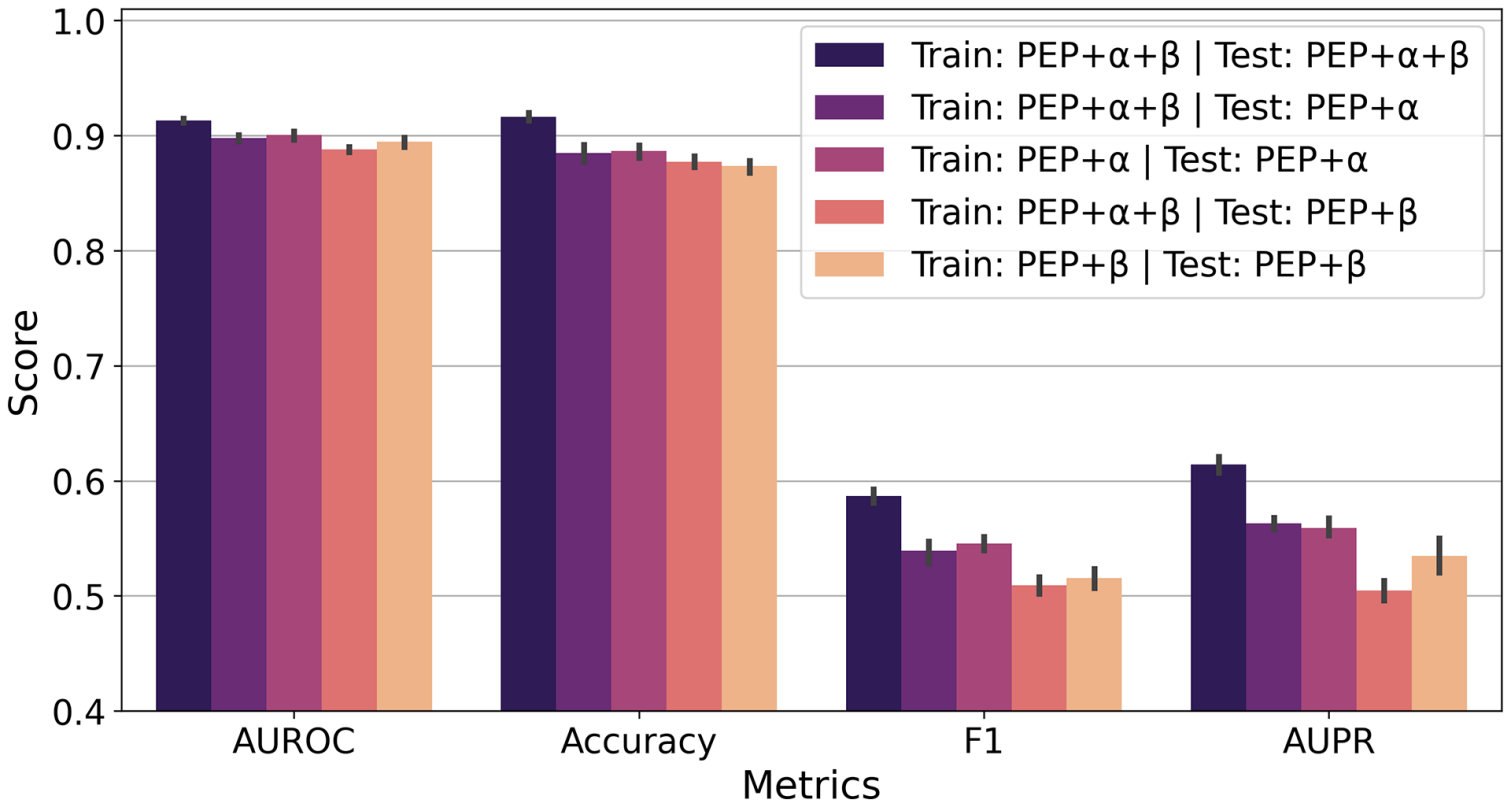
AVIB performance with missing input sequences. Confidence intervals are standard deviations over five repeated experiments on the α+β*set* with different independent training/test random splits. Train, training time sequences; Test, test time sequences

Figure 2 presents the experimental results. As expected, AVIB performance decreases when a CDR3 sequence is missing at test time. However, the performance achieved by AVIB when trained in the tri-sequence setting and tested on missing sequences is not consistently different to the performance deriving from a bi-sequence training. We only observe a significant difference in the AUPR score when the CDRα sequence is missing: AVIB trained on peptide+CDR3β achieves ∼3% higher AUPR than AVIB trained on peptide+CDR3α+CDR3β and tested with missing CDR3α.

### 3.6 OOD detection

Alemi *et al.* (2018) show that VIB has the ability to detect OOD samples. In this section, the OOD detection capabilities of AVIB are investigated. We assume that we have an in-distribution (ID) dataset $D^{ID}$ of $\left(x_{1}^{ID},\ldots ,x_{M}^{ID};y^{ID}\right)$ tuples, where $x_{i}$ denote input data sequences and *y* the class label. $D^{\mathrm{OOD}}$ denotes an OOD dataset of $\left(x_{1}^{\mathrm{OOD}},\ldots ,x_{M}^{\mathrm{OOD}};y^{\mathrm{OOD}}\right)$ tuples. We study the scenario in which the model only has access to ID samples $D_{\mathrm{train}}^{ID}$ at training time. The test set consists of $D_{\mathrm{test}}^{ID}\cup D_{\mathrm{test}}^{\mathrm{OOD}}$. We adopt the *Human TCR set* as ID dataset, i.e. $D^{ID}$. We perform experiments using the *Non-human TCR set* and the *Human MHC set* as OOD datasets, i.e. $D^{\mathrm{OOD}}$.

We leverage the expectation of the learned latent posterior conditioned on all input sequences and fit two class-conditional Gaussian distributions using the ID training samples, one for the binding samples and one for the non-binding ones (Equation 7). The class-conditional Gaussian distributions share the same covariance matrix (Equation 8). Analogously to Lee *et al.* (2018), we discriminate whether test samples are ID or OOD using the Mahalanobis distance score (AVIB-Maha) (Equation 9).

**Training and test sets for OOD detection.** Given a pair $\left(D^{ID},D^{\mathrm{OOD}}\right)$, we operate a random 80/20 training/test split of $D^{ID}$ into $D_{\mathrm{train}}^{ID}$ and $D_{\mathrm{test}}^{ID}$. We train AVIB on $D_{\mathrm{train}}^{ID}$ for TCR–peptide interaction prediction. No OOD samples are available at training time. We ensure that the number of ID and OOD samples in the test set is balanced by applying the procedure described in Supplementary Material S11.1. Experiments are repeated five times with different random training/test splits.

**Baselines.** For benchmark, we compare our results with several OOD detection methods: MSP (Hendrycks and Gimpel, 2016), ODIN (Liang *et al.*, 2017) and the AVIB rate (AVIB-R) $D_{KL}\left(p_{\Theta }(Z|x_{1:M}^{n})||r_{\omega }(Z)\right)$ (Alemi *et al.*, 2018). See Supplementary Material S11.2 for further details about the baseline methods.

**Evaluation metrics.** As evaluation metrics, in addition to AUROC and AUPR, we adopt the false positive rate at 95% true positive rate (FPR @ 95% TPR) and the detection error (see Supplementary Material S11.3).

**Fig. 3.**
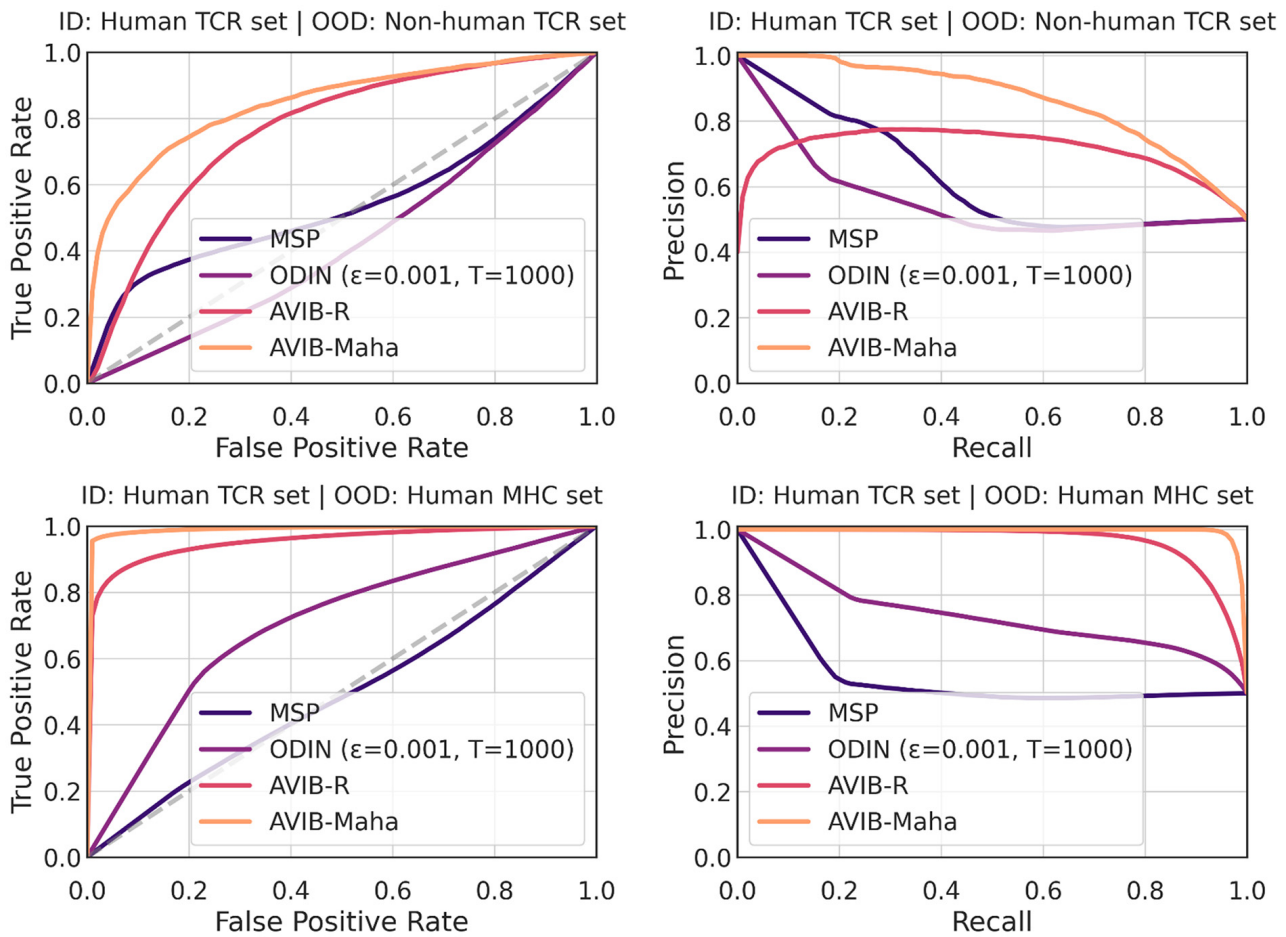
OOD detection—ROC and PR curves

Table 3 summarizes the OOD detection results for AVIB trained on the *Human TCR set* for TCR–peptide interaction prediction and using the *Non-human TCR set* and the *Human MHC set* as OOD datasets. Figure 3 shows the ROC and PR curves. AVIB-Maha achieves best results on all investigated metrics on both OOD datasets. On the *Non-human TCR set*, AVIB-Maha outperforms AVIB-R by ∼9% AUROC and >15% AUPR. On the *Human MHC set*, AVIB-Maha outperforms AVIB-R by ∼29% FPR at 95% TPR and ∼15% detection error.

**Table 3.** OOD detection results

*D* ^ID^/*D*^OOD^	Method	FPR at 95% TPR ↓	Detection error ↓	AUROC ↑	AUPR ↑
Human TCR/non-human TCR	MSP	0.962 ± 0.001	0.505 ± 0.001	0.540 ± 0.003	0.627 ± 0.003
	ODIN	0.962 ± 0.002	0.506 ± 0.001	0.425 ± 0.008	0.559 ± 0.014
	AVIB-R	0.719 ± 0.018	0.384 ± 0.009	0.768 ± 0.010	0.714 ± 0.011
	AVIB-Maha (ours)	**0.699 ± 0.011**	**0.374 ± 0.006**	**0.850 ± 0.002**	**0.871 ± 0.001**
Human TCR/human MHC	MSP	0.955 ± 0.002	0.503 ± 0.001	0.491 ± 0.007	0.550 ± 0.005
	ODIN	0.714 ± 0.047	0.382 ± 0.024	0.701 ± 0.029	0.763 ± 0.027
	AVIB-R	0.297 ± 0.027	0.174 ± 0.014	0.955 ± 0.004	0.964 ± 0.003
	AVIB-Maha (ours)	**0.006 ± 0.002**	**0.028 ± 0.001**	**0.994 ± 0.001**	**0.995 ± 0.001**

*Note*: Distinguishing in- and out-of-distribution test samples. *D*^ID^ is the in-distribution set; *D*^OOD^ is the out-of-distribution set, not available at training time. The reported confidence intervals are standard errors over five repeated experiments with different independent $D_{\mathrm{train}}^{ID}$/$D_{\mathrm{test}}^{ID}$ random splits. ↑ indicates larger value is better; ↓ indicates lower value is better. Best results are in bold. Baselines: MSP, ODIN and AVIB-R. For ODIN, the hyperparameters are ε=0.001 and *T *=* *1000, tuned on a validation set.

## 4 Conclusion

In this article, we propose AVIB, a multi-sequence generalization of the Variational Information Bottleneck (Alemi *et al.*, 2016), which uses AoE to implicitly approximate the posterior distribution over latent encodings conditioned on multiple input sequences. We apply AVIB to the TCR–peptide interaction prediction problem, a fundamental challenge in immuno-oncology. We show that our method significantly improves on the state-of-the-art baselines ERGO II (Springer *et al.*, 2021) and NetTCR-2.0 (Montemurro *et al.*, 2021). We demonstrate the effectiveness of AoE with a benchmark against PoE, as well as with an ablation study. We also show that AoE achieves the best results on peptide-MHC binding affinity regression. Furthermore, we demonstrate that AVIB can handle missing data sequences at test time. We then leverage the bottleneck posterior distribution learned by AVIB and demonstrate that it can be used to effectively detect OOD amino acid sequences. Our method significantly outperforms the baselines MSP (Hendrycks and Gimpel, 2016), ODIN (Liang *et al.*, 2017) and AVIB-R (Alemi *et al.*, 2018). Interestingly, we observe that generalization to unseen sequences remains a challenging problem for all investigated models. These results are analogous to those of Grazioli *et al.* (2022b). We believe this drop in performance is due to the sparsity of the observed training sequences. Future work should focus on tackling the problem of generalization by, for example, simulating or approximating the chemical interactions of TCR and peptides (or pMHCs), as well as their 3D structures.

*Financial Support*: none declared.

*Conflict of Interest*: none declared.

## Supplementary Material

btac820_Supplementary_Data
