# Supplementary material for: Attentive Variational Information Bottleneck for TCR–peptide interaction prediction
Source: Bioinformatics. 2022 Dec 26;39(1):btac820. doi: 10.1093/bioinformatics/btac820 (PMC9825246; doi:10.1093/bioinformatics/btac820)
Supplement: btac820_Supplementary_Data [file btac820_supplementary_data.zip › btac820_Supplementary_Data/supplementary-final-cropped.pdf]

1 TCR-pMHC Complex

Figure 4 depicts the TCR-pMHC complex. The TCR α-chain and β-chain are depicted in purple and blue, respectively. The MHC class I is composed of an α-chain (dark green) and β_2-microglobulin (orange). The peptide (light green) is visible between TCR and MHC. The peptide is presented by a cell through the MHC and recognized by the TCR, which allows a T cell to distinguish between healthy and infected or cancerous cells.

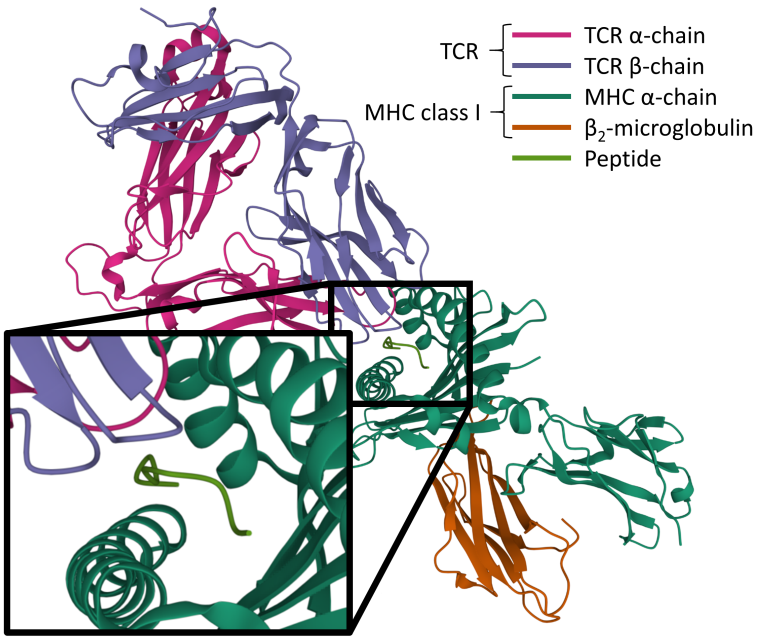

Fig. 4. TCR-pMHC complex. Figure created using Mol* Viewer (Sehnal et al., 2021).

2 Multi-head Attention

In this section, we report the equations of the multi-head attention block as defined in Vaswani *et al.* (2017), which is employed by AoE.

First, the "Scaled Dot-Product Attention" is defined as:

$$Attention(\boldsymbol{Q}, \boldsymbol{K}, \boldsymbol{V}) = softmax(\frac{\boldsymbol{Q}\boldsymbol{K}^T}{\sqrt{d_k}})\boldsymbol{V}. \tag{10}$$

$\boldsymbol{Q}$, $\boldsymbol{K}$ and $\boldsymbol{V}$ are input matrices, which stack a set of queries, keys and values, respectively. Queries and keys are vectors of dimension d_k. Values are vectors of dimension d_v.

(Vaswani *et al.*, 2017) found it beneficial to linearly project the queries, keys and values h times with different, learned linear projections to d_k, d_k and d_v dimensions, respectively, assuming d_{model} is the initial dimension of the queries, keys and values before this projection. The multi-head attention is defined as:

$$MultiHead(\boldsymbol{Q}, \boldsymbol{K}, \boldsymbol{V}) = Concat(head_1, ..., head_h)\boldsymbol{W}^O, \tag{11}$$

where $head_i = Attention(\boldsymbol{Q}\boldsymbol{W}_i^Q, \boldsymbol{K}\boldsymbol{W}_i^K, \boldsymbol{V}\boldsymbol{W}_i^V)$. The projection matrices are $\boldsymbol{W}_i^Q \in \mathbb{R}^{d_{model} \times d_k}$, $\boldsymbol{W}_i^K \in \mathbb{R}^{d_{model} \times d_k}$, $\boldsymbol{W}_i^V \in \mathbb{R}^{d_{model} \times d_v}$ and $\boldsymbol{W}^O \in \mathbb{R}^{hd_v \times d_{model}}$.

In our work, since we apply self-attention, $\boldsymbol{Q} = \boldsymbol{K} = \boldsymbol{V}$ and $d_k = d_v = d_Z$, where d_Z is the bottleneck dimension.

3 Implementation Details and Training Setup

In this work, there are three data sequences related to TCR-peptide interaction prediction: $\boldsymbol{x}_{Peptide}$, $\boldsymbol{x}_{CDR3\beta}$, and $\boldsymbol{x}_{CDR3\alpha}$. Additionally, for peptide-MHC binding prediction, we consider $\boldsymbol{x}_{MHC}$, i.e. the MHC amino acids pseudo-sequence. For each sequence, the dedicated stochastic encoder has the form $\widetilde{q}_{\boldsymbol{\theta}}(Z|\boldsymbol{x}) = \mathcal{N}(f_e^{\mu}(\boldsymbol{x}), f_e^{\sigma}(\boldsymbol{x}))$. f_e^{μ} and f_e^{σ} are the two output branches of the neural stochastic encoder depicted in Figure 5.

The neural stochastic encoder of Figure 5 presents an architecture which is strongly inspired by NetTCR-2.0 (Montemurro *et al.*, 2021). It operates 1D convolutions of the BLOSUM50-encoded peptides with kernel sizes 1, 3, 5, 7 and 9. After the convolutions, 1D max pooling is operated, followed by ReLu activation functions. The obtained vectors are then concatenated. Eventually, two parallel fully-connected layers output two vectors of size

d_Z for $\boldsymbol{\mu}$ and $\boldsymbol{\sigma}$. d_Z is the size of the bottleneck, i.e. the dimension of Z. We set $d_Z = 150$ after a fine-tuning on a validation set over the values $\{50, 100, 150, 200\}$. For a more stable computation, we let $f_e^\sigma(\boldsymbol{x})$ model the logarithm of the variance $\log \boldsymbol{\sigma}^2$.

The decoder consists in a simple neural network with 3 fully-connected layers and ReLu activations. This implements the binary classification for TCR-peptide interaction prediction or regression for peptide-MHC binding prediction.

$r_{\boldsymbol{\omega}}(Z)$ (Equation 6) and the latent prior $p(Z)$ are treated as d_Z-dimensional spherical Gaussian distributions. Analogously to Alemi *et al.* (2016), we set $r_{\boldsymbol{\omega}}(Z) = p(Z) = \mathcal{N}(\mathbf{0}, \boldsymbol{I})$. β is set to 10^{-6} after a fine-tuning on a validation set over the values $\{0, 10^{-8}, 10^{-6}, 10^{-4}, 10^{-2}, 1\}$.

The networks are trained using the Adam optimizer with a learning rate of 10^{-3} and a L_2 weight decay with $\lambda = 10^{-5}$. The batch size is set to 4,096. 0.3 drop-out is used at training time. For the multi-head attention module in AoE, we use 5 heads. Cosine annealing learning rate scheduler with a period of 10 epochs is adopted. For learning rate, L_2 weight decay λ, batch size, number of heads in AoE, and learning rate scheduler we do not perform extensive hyperparameter tuning. The training is performed for 200 epochs and, in order to avoid over-fitting, the best model is selected by saving the weights corresponding to the epoch where the AUROC is maximum on the validation set. The validation set is obtained via 80/20 stratified random split of the training set. Training and test sets are obtained via a non-stratified 80/20 split of the whole dataset. Experiments are repeated 5 times with different training/test splits to ensure unbiased performance evaluation.

All experiments are performed on a CentOS Linux 8 machine with NVIDIA GeForce RTX 2080 Ti GPUs and CUDA 10.2 installed. Algorithms are implemented in Python 3.6 using PyTorch (Paszke *et al.*, 2019) version 1.10.

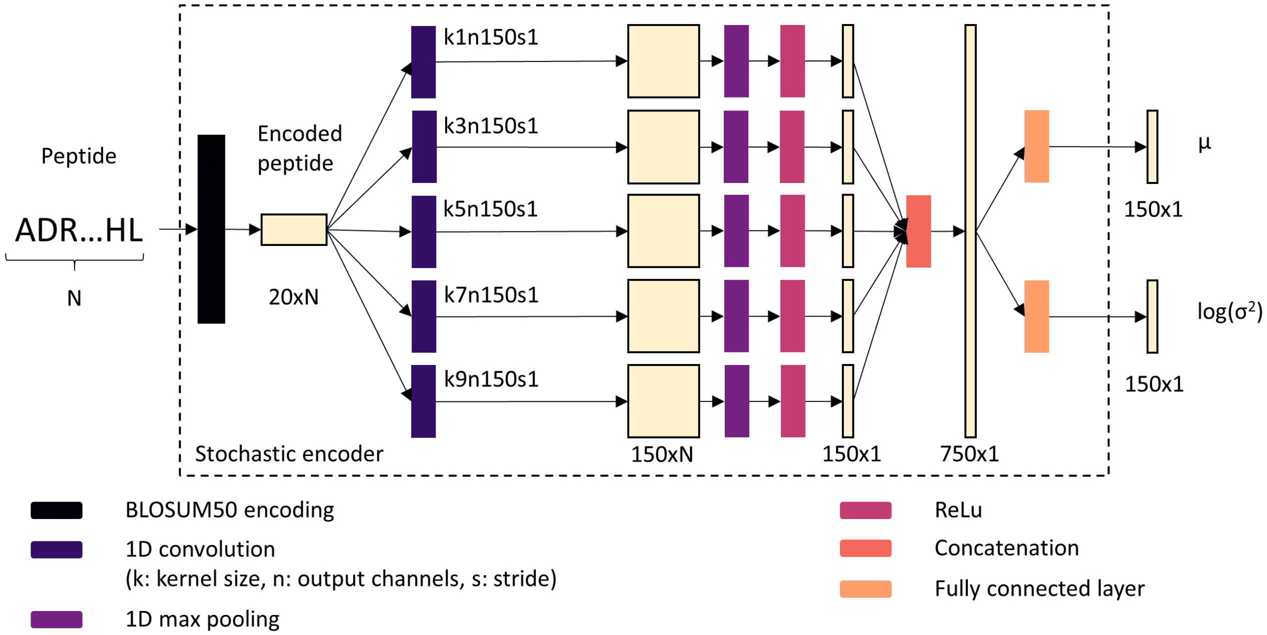

Fig. 5. **Architecture of the stochastic encoders for amino acid sequences.**

4 AVIB Architecture

Figure 6 provides a schematic depiction of the architecture of AVIB in the tri-sequence setting. Inputs are peptide, CDR3β, and CDR3α.

5 Datasets Details

Figure 7 depicts the distributions of the human TCR data for both the α+β *set* and the β *set*. The two datasets have similar peptide distributions, but mostly contain different CDR3β sequences.

5.1 Amino Acid Sequences Length Distribution

Figure 8 depicts the amino acids sequence length distributions for the datasets used in this work. The length consists in the number of amino acids which constitute the peptides, CDR3α, CDR3β, and MHC pseudo-sequences. In this work, all these types of molecules are represented as sequences of amino acids.

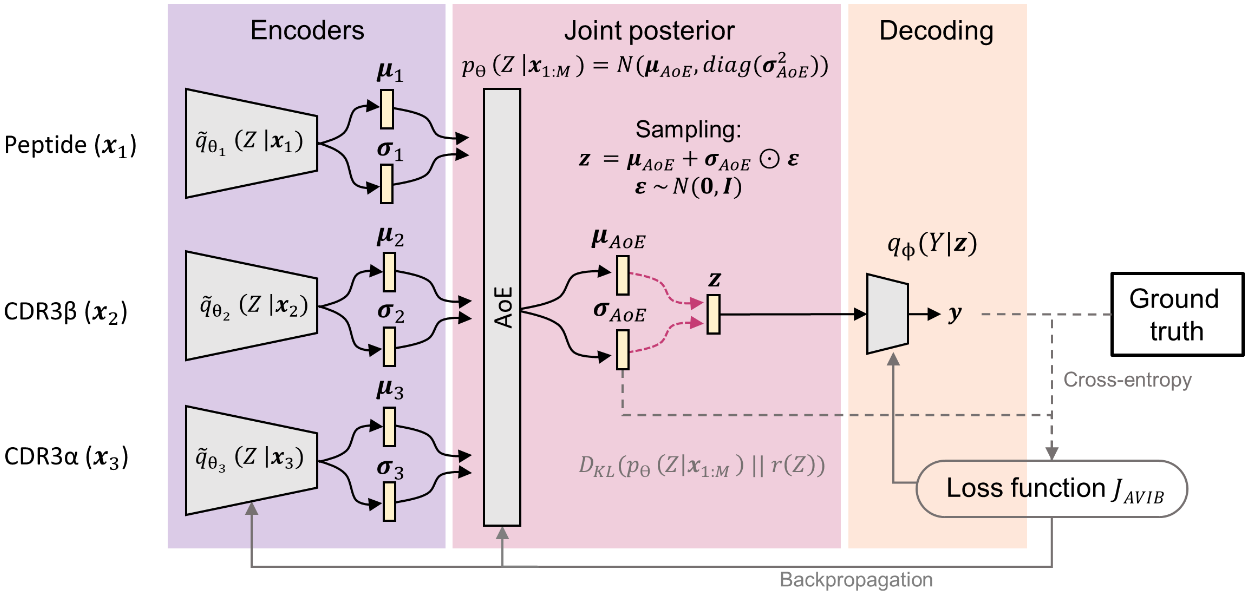

Fig. 6. **Tri-sequence AVIB architecture.**

5.2 Class Distribution

Figure 9 depicts the class distributions of the β *set* and α+β *set*. We do not report the class distributions of *Non-human TCR set* and *Human MHC set*, as they are only used for OOD detection experiments and not for TCR-peptide interaction prediction.

It is possible to observe that the TCR data presents many more non-binding samples (0), compared to binding ones (1). We handle this class imbalance by means of balanced batch sampling, i.e. when we sample a batch at training time, we ensure that the numbers of binding and non-binding samples are equal.

5.3 Binding Affinity Distribution

Figure 10 presents the BA distribution of the *NetMHCIIpan-4.0 set*. It can be observed that the dataset is slightly unbalanced towards (peptide, MHC) pairs with BA < 0.5.

6 Full Multi-sequence Objective

The J_{AVIB} objective of Equation 6 assumes that all M data sequences are present. Let us assume we train AVIB in the tri-sequence setting, considering $(\boldsymbol{x}_{Peptide}, \boldsymbol{x}_{CDR3\alpha}, \boldsymbol{x}_{CDR3\beta})$ triples as input. Optimizing Equation 6 has the unfortunate consequence of not training AVIB for handling missing data sequences. In fact, if AVIB is trained on peptide, CDR3α and CDR3β data, it is desirable to also perform inference in situations where only two sequences are available, e.g. peptide and CDR3α, or peptide and CDR3β [6]. This is a common real-world setting and it would be unpractical to train three different models, one for each combination of peptide and CDR3s.

In order to circumvent this limitation and allow AVIB to work at test time with a missing CDR3 sequence, we need to compute the AVIB objective for all possible combinations of peptide and CDR3s. Hence, by defining $J_{AVIB}(\boldsymbol{x}_k, ..., \boldsymbol{x}_l)$ as Equation 6 restricted to the $k^{th}, ..., l^{th}$ sequences, the full multi-sequence objective is:

$$J_{AVIB}(\boldsymbol{x}_{Peptide}, \boldsymbol{x}_{CDR3\alpha}) + J_{AVIB}(\boldsymbol{x}_{Peptide}, \boldsymbol{x}_{CDR3\beta}) + J_{AVIB}(\boldsymbol{x}_{Peptide}, \boldsymbol{x}_{CDR3\alpha}, \boldsymbol{x}_{CDR3\beta}) \,. \tag{12}$$

In this work, in all tri-sequence TCR-peptide experiments we adopt Equation 12.

7 Peptide+CDR3α Binding Prediction Results

Table 4 presents TCR-peptide interaction prediction results computed on the α+β *set* in the bi-sequence setting, with peptide and CDR3α sequence. Considering AVIB performance on the α+β *set* in the various multi-sequence settings (see Table 1 and Table 4), we observe that best results are achieved in the tri-sequence (peptide+CDR3α+CDR3β) case. This shows that jointly observing CDR3α and CDR3β sequences is beneficial for TCR-peptide

[6] As we investigate TCR-peptide interaction prediction, we assume the peptide sequence is always present.

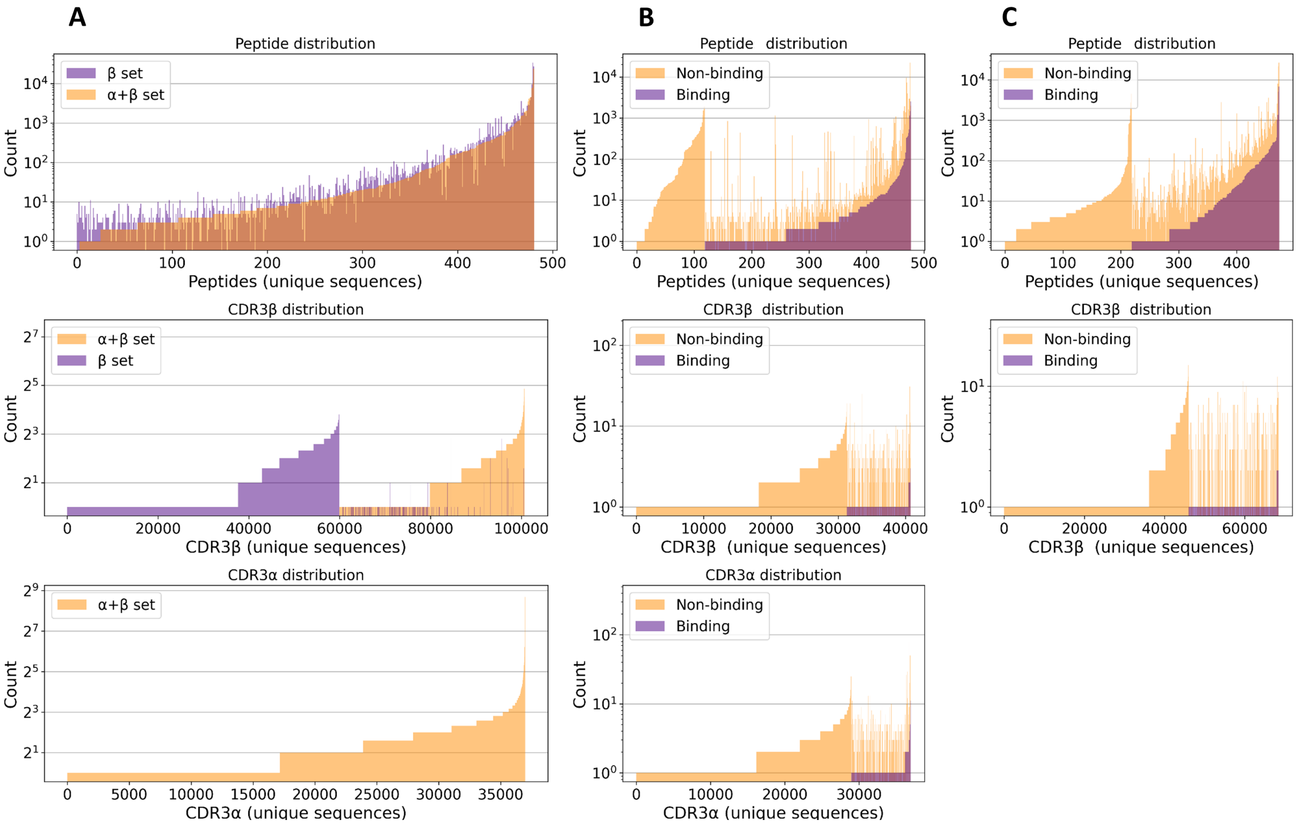

Fig. 7. TCR-peptide datasets distributions. A point on the x-axis represents one unique sequence of amino acids. The y-axis represents how many samples present that specific sequence. Samples are sorted by count. (A) Distributions of the α+β *set* and β *set* considering positive and negative samples jointly. (B) Class-specific distributions of the α+β *set*. (C) Class-specific distributions of the β *set*.

Table 4. **TCR-peptide interaction prediction results - Peptide+CDR3α.** The reported confidence intervals are standard errors over 5 repeated experiments with different independent training/test random splits. Reported scores are computed on the test sets. PEP: peptide; α: CDR3α sequence.

Dataset	Inputs	Method	AUROC	AUPR	F1
α+β *set*	Pep+α	AVIB (ours)	0.900 ± 0.001	0.559 ± 0.002	0.546 ± 0.001

interaction prediction. Additionally, this demonstrates that AVIB is an effective multi-sequence learning method, as the tri-sequence results outperform both peptide+CDR3α and peptide+CDR3β results.

8 Cross-dataset Generalization Experiments

In order to provide a thorough evaluation of AVIB's performance, we train and test the model in a cross-dataset setting. Considering only peptide and CDR3β, we first train on the α+β *set* and then test on the β *set*. This experiment is analogous to the TPP-II test presented by Springer *et al.* (2021).

Figure 11 depicts the cross-dataset generalization results. For benchmark, we compare against ERGO II, NetTCR-2.0 and LUPI-SVM. In Figure 11, it can be observed that all tested models provide approximately random predictions.

We hypothesize that the lack of cross-dataset generalization lays in the distribution of the CDR3β sequences: although the α+β *set* and the β *set* share common peptides, the two sets mostly present different CDR3β sequences (see Figure 7). This appears to be an important limitation of current machine-learning-based prediction models for TCR-peptide binding. Robust generalization on unseen CDR3s is an important prerequisite for real-world applicability of this class of models.

9 Visualization of the Attention Weights

As explained in the manuscript, AVIB makes use of multi-head self-attention. Each head calculates attention weights, which we can average to obtain a unique attention weight matrix. To interpret these attention weights, we select multiple true positive samples (i.e. binding) from the β *set* and compute the

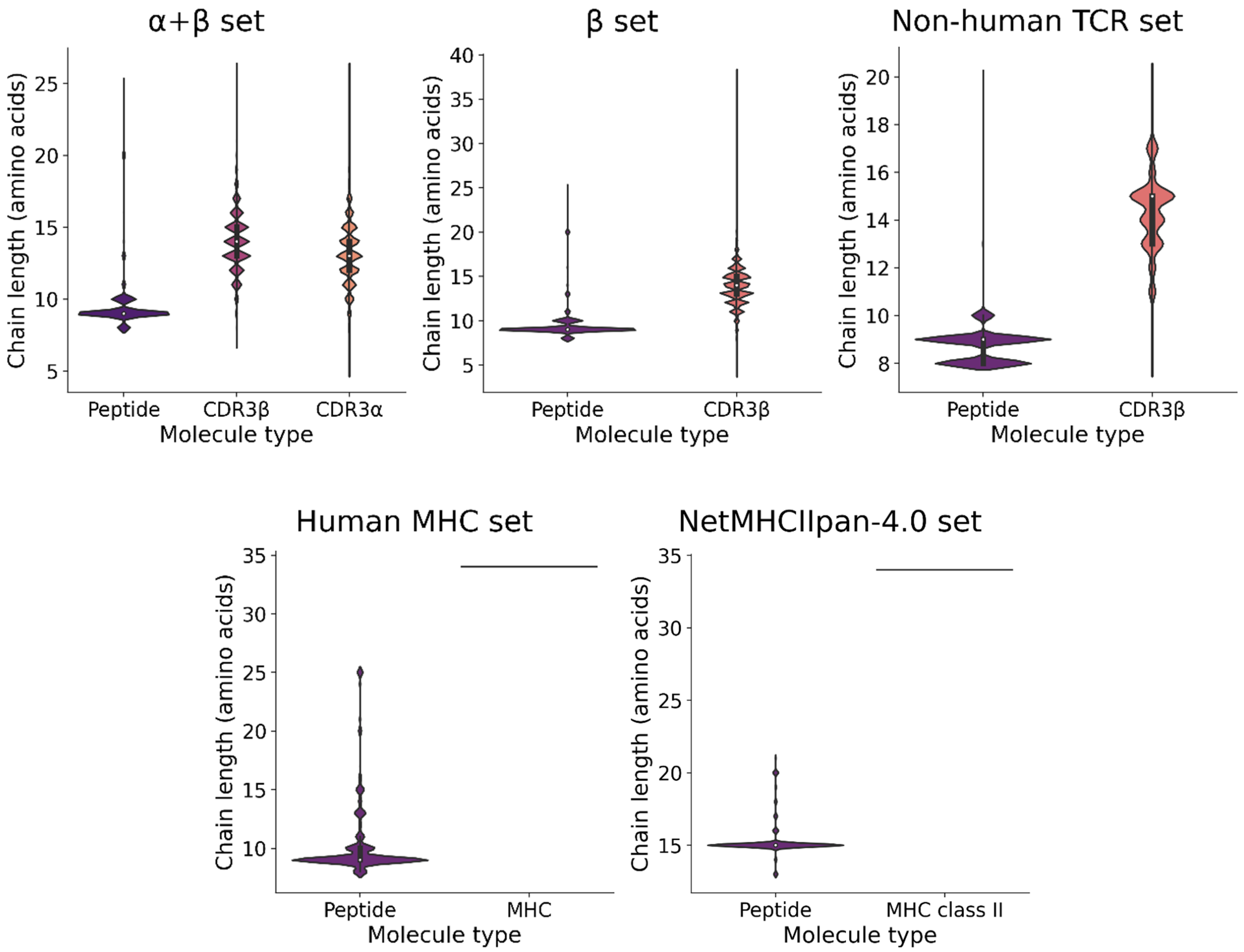

Fig. 8. Length distributions of the amino acid sequences for all datasets.

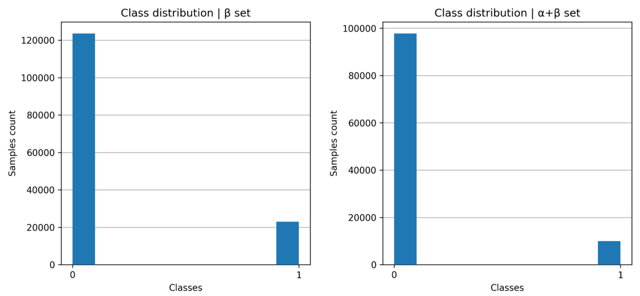

Fig. 9. Class distribution of α+β ***set*** **and** β ***set*.**

0: non-binders; 1: binders.

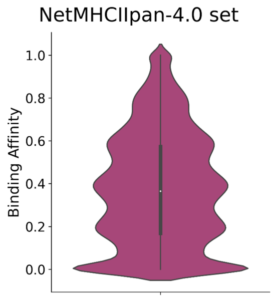

Fig. 10. Binding affinity distribution of ***NetMHCIIpan-4.0 set*.**

average attention weights from the block which estimates $\boldsymbol{\mu}_{AoE}$. Here, we visualize the attention weights as a 3x3 matrix, representing the relationships between $\boldsymbol{\mu}_{CDR3\beta}$, $\boldsymbol{\mu}_{Peptide}$ and the non-informative prior $\boldsymbol{\mu}_0 = \mathbf{0}$.

We simulate mutations to disrupt the peptide sequence and therefore the binding affinity, so as to visualize the effect of mutations on the predicted attention weights. We choose mutations that *should* disrupt the interactions. First, at position two of the peptide, we identify one of the most unlikely mutation using BLOSUM50, which should disrupt the interaction as it is involved in the formation of the binding complex Szeto *et al.* (2020). Then, we additionally mutate a central residue to Proline, so as to change the dihedral angle of the peptide, thus further disrupting the interaction.

As shown in Supplementary Figure 12, we see that progressively disrupting the peptide leads to a gradual drop in the predicted binding probability. Additionally, the peptide-CDR3β attention weight tends to decrease, while the CDR3β-peptide attention weight tends to rise.

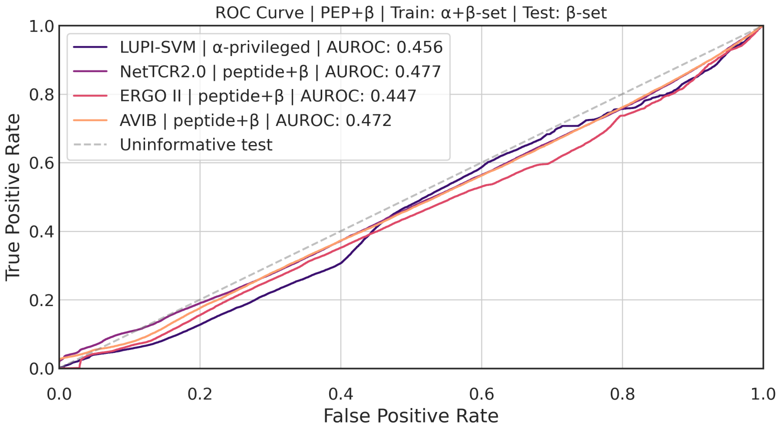

Fig. 11. Cross-dataset generalization results. Models are trained on the α+β *set* and tested on the β *set*. For both training and testing, the peptide and the CDR3β are employed.

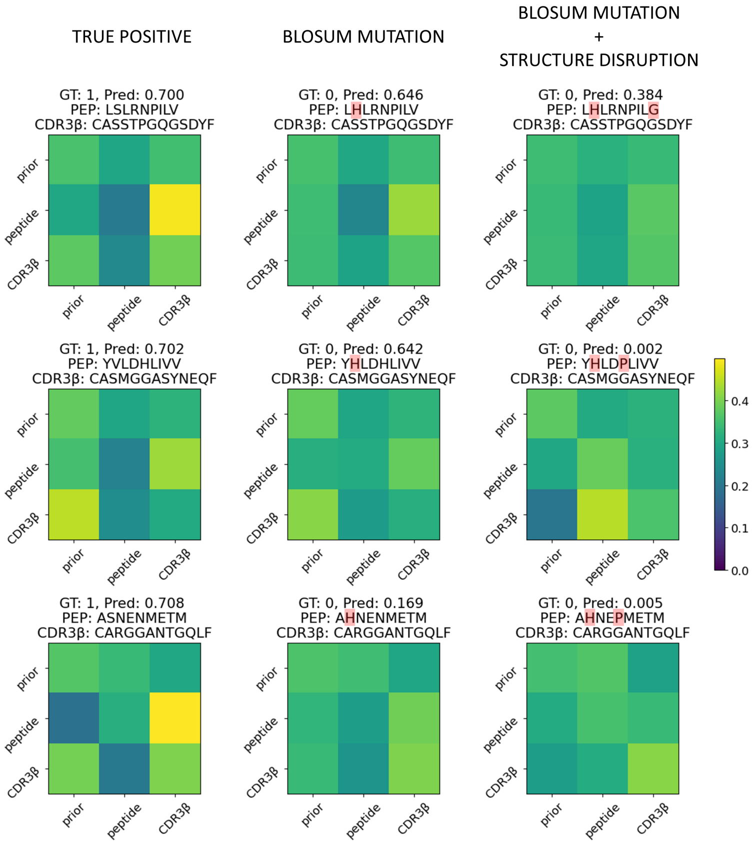

Fig. 12. Attention weights visualization - peptide+CDR3β setting. In the first column, we display true positive samples from the β *set*. In the second column, we simulate an unlikely mutation of the second residue using the BLOSUM50 matrix. In the third column, we additionally mutate a central residue to Proline, which should impact the dihedral angle and therefore the 3D structure of the peptide. Mutated residues are highlighted in red. GT: ground-truth label. Pred: AVIB prediction. PEP: peptide.

10 Additional Ablation Results

In Table 5, we report ablation results using MaxPOOLoE.

Table 5. **Multi-sequence posterior approximation - ablation with MaxPOOLoE.** TCR-peptide binding prediction experiments are performed on the α+β *set*. Peptide-MHC BA regression experiments are performed on the *NetMHCIIpan-4.0 set*. Confidence intervals are standard errors over 5 repeated experiments with different training/test random splits. PEP: peptide; α: CDR3α sequence; β: CDR3β sequence; MHC II: MHC class II pseudo-sequence. Best results are in **bold**; ↑ indicates larger value is better; ↓ indicates lower value is better.

Inputs	Metric	↑ / ↓	MaxPOOLoE
Pep+β	AUROC	↑	0.886 ± 0.001
	AUPR		0.509 ± 0.002
	F1		0.475 ± 0.001
	Accuracy		0.841 ± 0.001
Pep+α+β	AUROC	↑	0.905 ± 0.001
	AUPR		0.569 ± 0.003
	F1		0.530 ± 0.003
	Accuracy		0.874 ± 0.002
Pep+MHC II	MSE	↓	0.0362 ± 0.0001
	RMSE		0.150 ± 0.001
	R^2	↑	0.466 ± 0.002

11 Out-of-distribution Detection

11.1 Construction of the Test Set

The test set is constituted as follows. If $|\mathcal{D}^{OOD}| > |\mathcal{D}^{ID}_{test}|$, we sample $\mathcal{D}^{OOD}_{test}$ from $\mathcal{D}^{OOD}$ without replacement so that $|\mathcal{D}^{OOD}_{test}| = |\mathcal{D}^{ID}_{test}|$; $\mathcal{D}^{ID}_{test} \cup \mathcal{D}^{OOD}_{test}$ is the final test set. If $|\mathcal{D}^{OOD}| \leq |\mathcal{D}^{ID}_{test}|$, we sample $\mathcal{D}^{ID}_{test*}$ from $\mathcal{D}^{ID}_{test}$ without replacement so that $|\mathcal{D}^{ID}_{test*}| = |\mathcal{D}^{OOD}|$; $\mathcal{D}^{ID}_{test*} \cup \mathcal{D}^{OOD}$ is the final test set. This guarantees that the number of ID and OOD samples in the test set is balanced.

11.2 Out-of-distribution Detection Baselines

In the following, we describe various techniques for detecting OOD samples using neural networks. We use these methods as baseline and benchmark against them.

Maximum over softmax probabilities. A popular baseline for OOD detection consists in using the maximum softmax probability (MSP) as a confidence score, i.e. $score_{MSP}(\boldsymbol{x}) = \max_c p(y = c|\boldsymbol{x})$, where c represents a class label (Hendrycks and Gimpel, 2016). This method is based on the assumption that OOD samples will lead to less confident predictions. In practice, this is not always true.

ODIN. ODIN was first proposed to detect OOD images with neural networks (Liang *et al.*, 2017) and is based on the observation that using temperature scaling and adding small perturbations to the input can separate the softmax score distributions between ID and OOD samples. Let $f(\boldsymbol{x})$ denote the pre-softmax logits of a neural network. Given a temperature $T \in \mathbb{R}^+$, ODIN computes a temperature-scaled softmax score for each class $c = 1, ..., K$: $S_c(\boldsymbol{x}; T) = \frac{exp(f_c(\boldsymbol{x})/T)}{\sum_{c=1}^{K} exp(f_c(\boldsymbol{x})/T)}$. By defining $S_{\hat{y}}(\boldsymbol{x}; T) = \max_c S_c(\boldsymbol{x}; T)$, small perturbations of the inputs are computed as $\tilde{\boldsymbol{x}} = \boldsymbol{x} - \varepsilon sign(-\nabla_{\boldsymbol{x}} log S_{\hat{y}}(\boldsymbol{x}; T))$, where ε is the perturbation magnitude. The ODIN score is then defined as $score_{ODIN}(\boldsymbol{x}) = \max_c p(y = c|\tilde{\boldsymbol{x}}; T)$.

VIB. Alemi *et al.* (2018) show that VIB - despite not being explicitly designed for OOD detection - gives two natural information theoretic metrics for uncertainty quantification: the rate and the entropy of the classifier. The rate is the KL divergence between the conditional distribution over latent encodings given an input $\boldsymbol{x}$ and the encodings space defined by the learned marginal $D_{KL}\left(p_{\boldsymbol{\theta}}(Z|\boldsymbol{x})||r_{\boldsymbol{\omega}}(Z)\right)$. The entropy of the classifier is $H(Y|Z)$. In our experiments, as our problem is multi-sequence, we compute the AVIB rate (AVIB-R): $D_{KL}\left(p_{\boldsymbol{\Theta}}(Z|\boldsymbol{x}^n_{1:M})||r_{\boldsymbol{\omega}}(Z)\right)$.

ODIN and Mahalanobis distance can virtually work with any pre-trained neural model and do not require OOD samples at training time. However, in practice, experimental results (Liang *et al.*, 2017; Lee *et al.*, 2018) show that tuning T and ε for ODIN and ε and the α coefficients for the Mahalanobis distance strongly affects these methods' performance. Hence, the knowledge of a set of OOD validation samples to fit these parameters is in fact needed.

11.3 FPR @ 95% TPR and Detection Error

In this section, we describe two metrics used for evaluating OOD detection, the false positive rate at 95% true positive rate (FPR @ 95% TPR) and the detection error.

- FPR @ 95% TPR: the probability that a positive (ID) example is misclassified as negative (OOD), i.e. the false positive rate, when the true positive rate is as high as 95%;
- Detection error (P_e): it measures the misclassification probability when TPR is 95%. It is defined as $P_e = 0.5(1 - TPR) + 0.5FPR$, assuming that both ID and OOD examples have the equal probability of appearing in the test set.

For evaluating OOD detection, we use these two metrics as well as AUPR and AUROC.
