# Supplementary figures and images for: Attentive Variational Information Bottleneck for TCR–peptide interaction prediction
Source: Bioinformatics. 2022 Dec 26;39(1):btac820. doi: 10.1093/bioinformatics/btac820 (PMC9825246; doi:10.1093/bioinformatics/btac820)

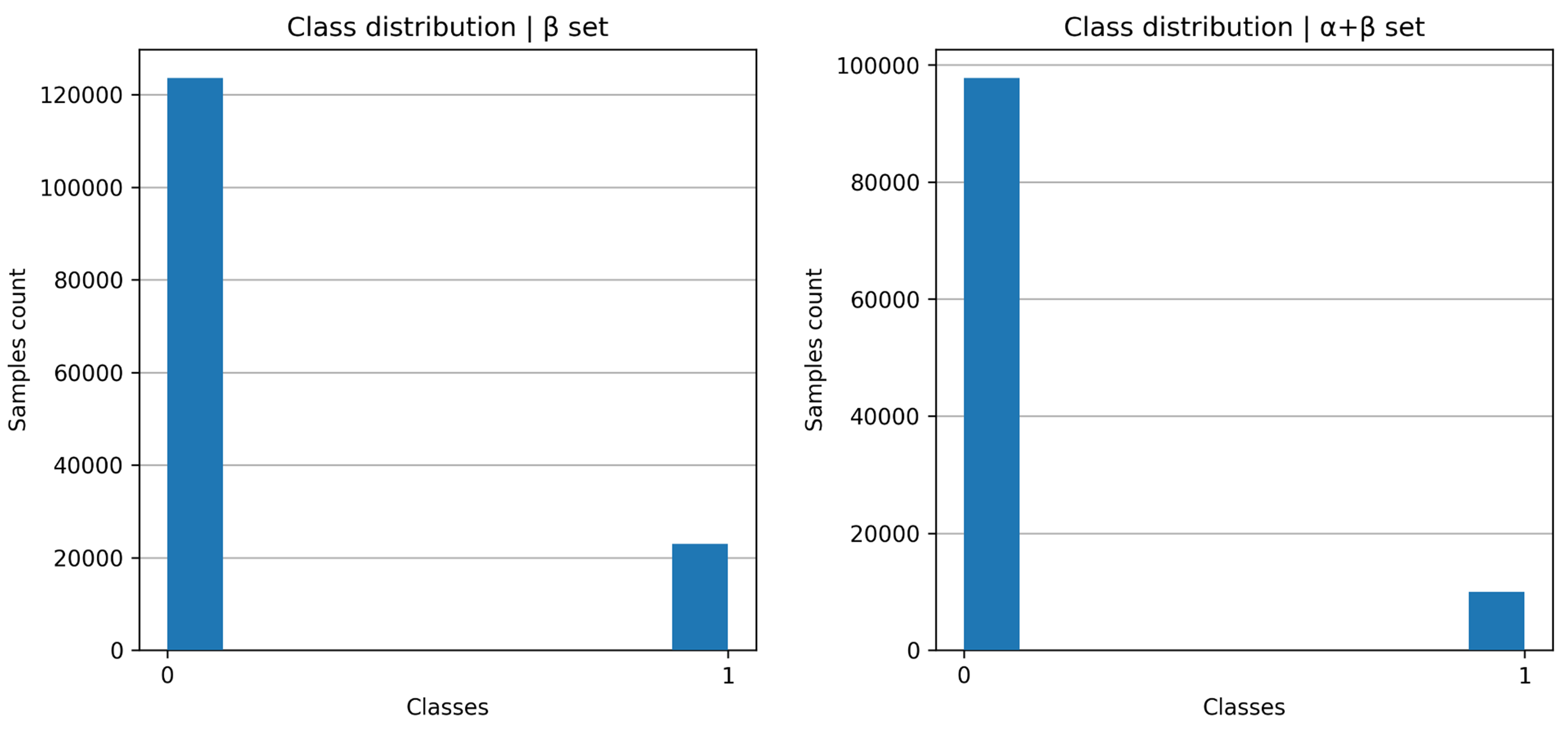

Supplement: btac820_Supplementary_Data [file btac820_supplementary_data.zip › btac820_Supplementary_Data/Sub-Fig-6.png]

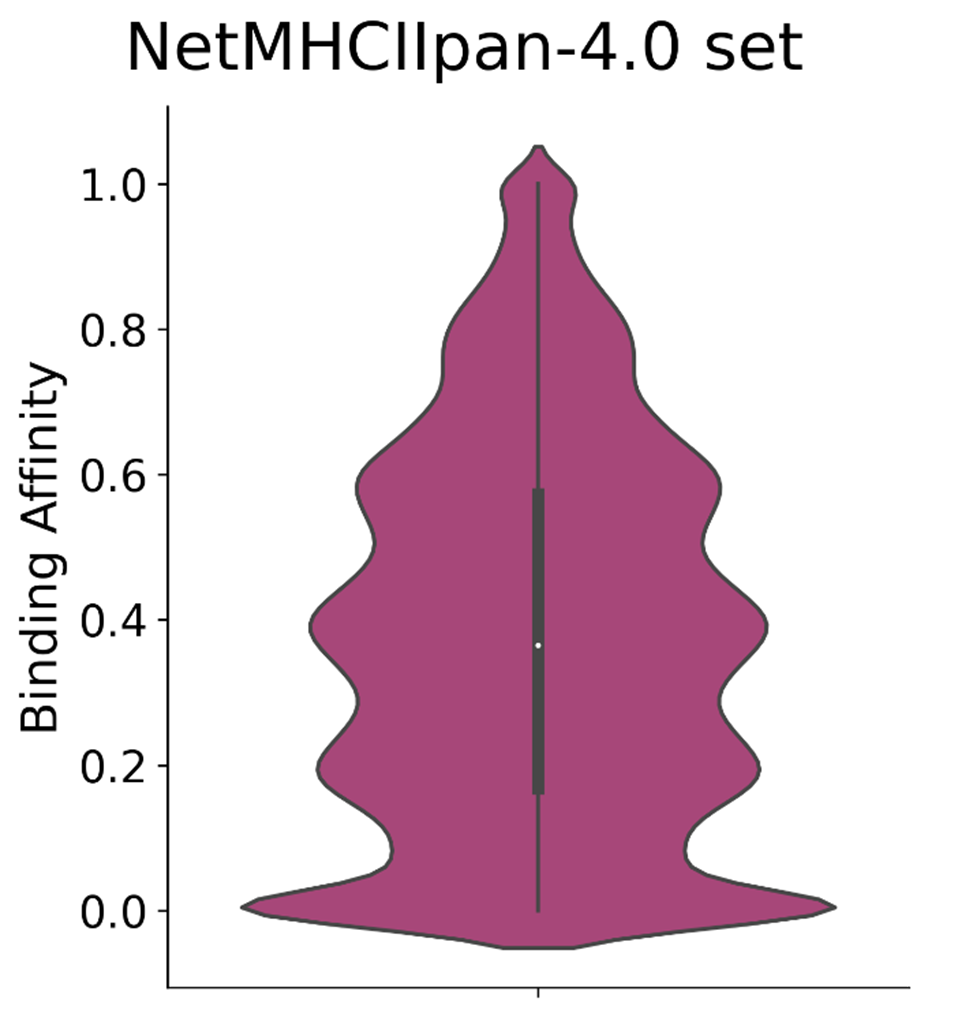

Supplement: btac820_Supplementary_Data [file btac820_supplementary_data.zip › btac820_Supplementary_Data/Sub-Fig-7.png]

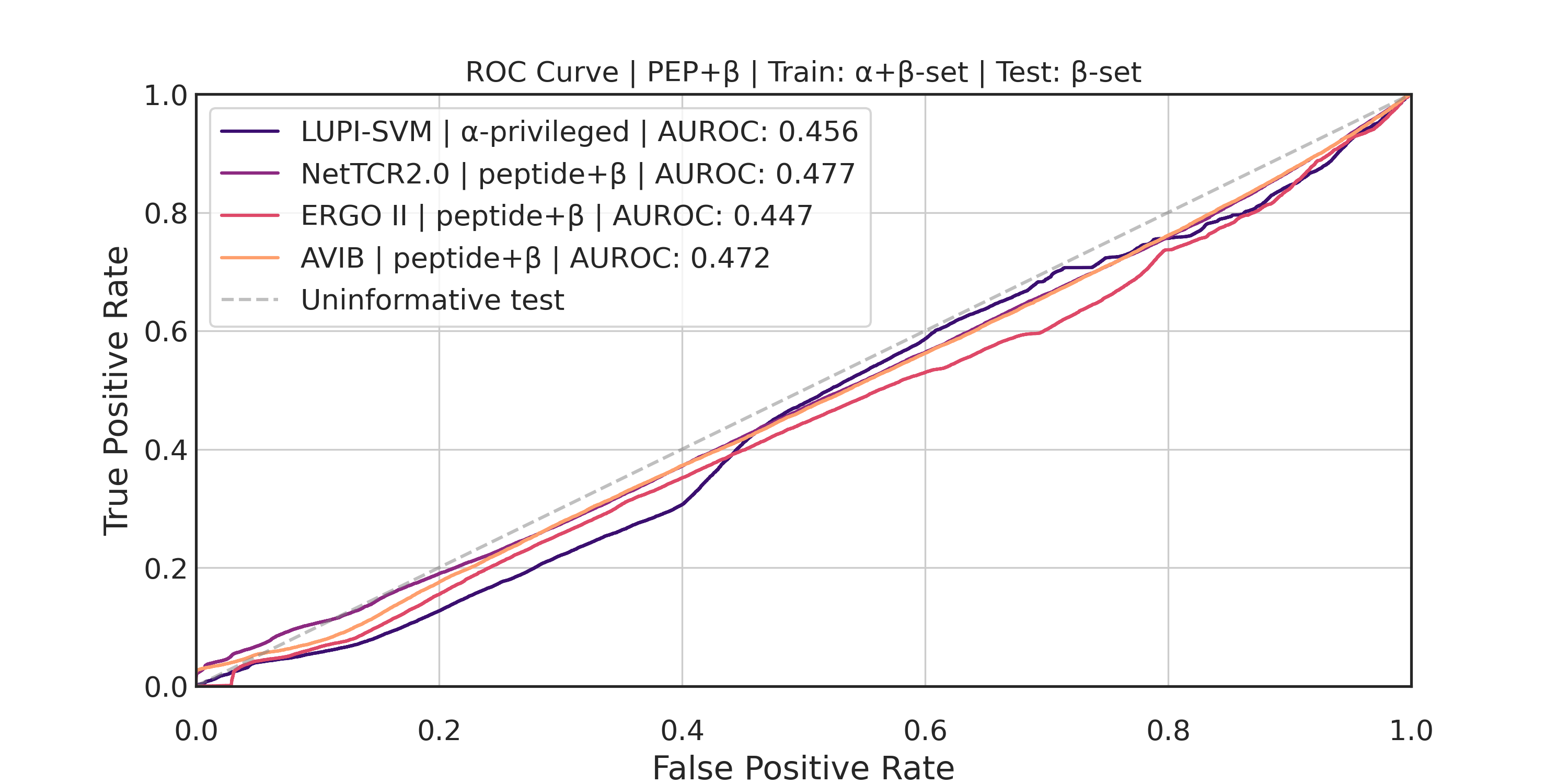

Supplement: btac820_Supplementary_Data [file btac820_supplementary_data.zip › btac820_Supplementary_Data/Sub-Fig-8.png]

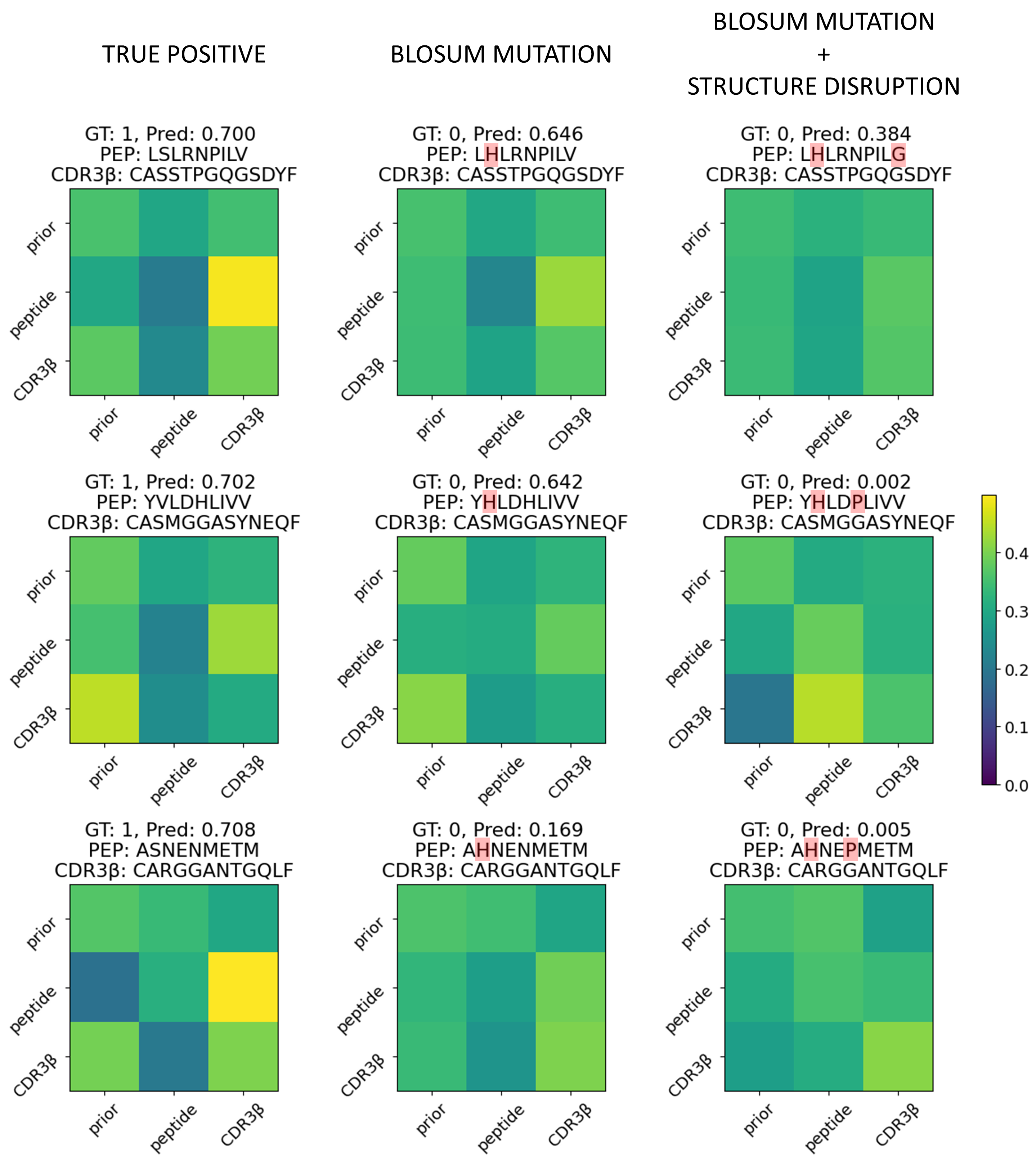

Supplement: btac820_Supplementary_Data [file btac820_supplementary_data.zip › btac820_Supplementary_Data/Sub-Fig-9.png]

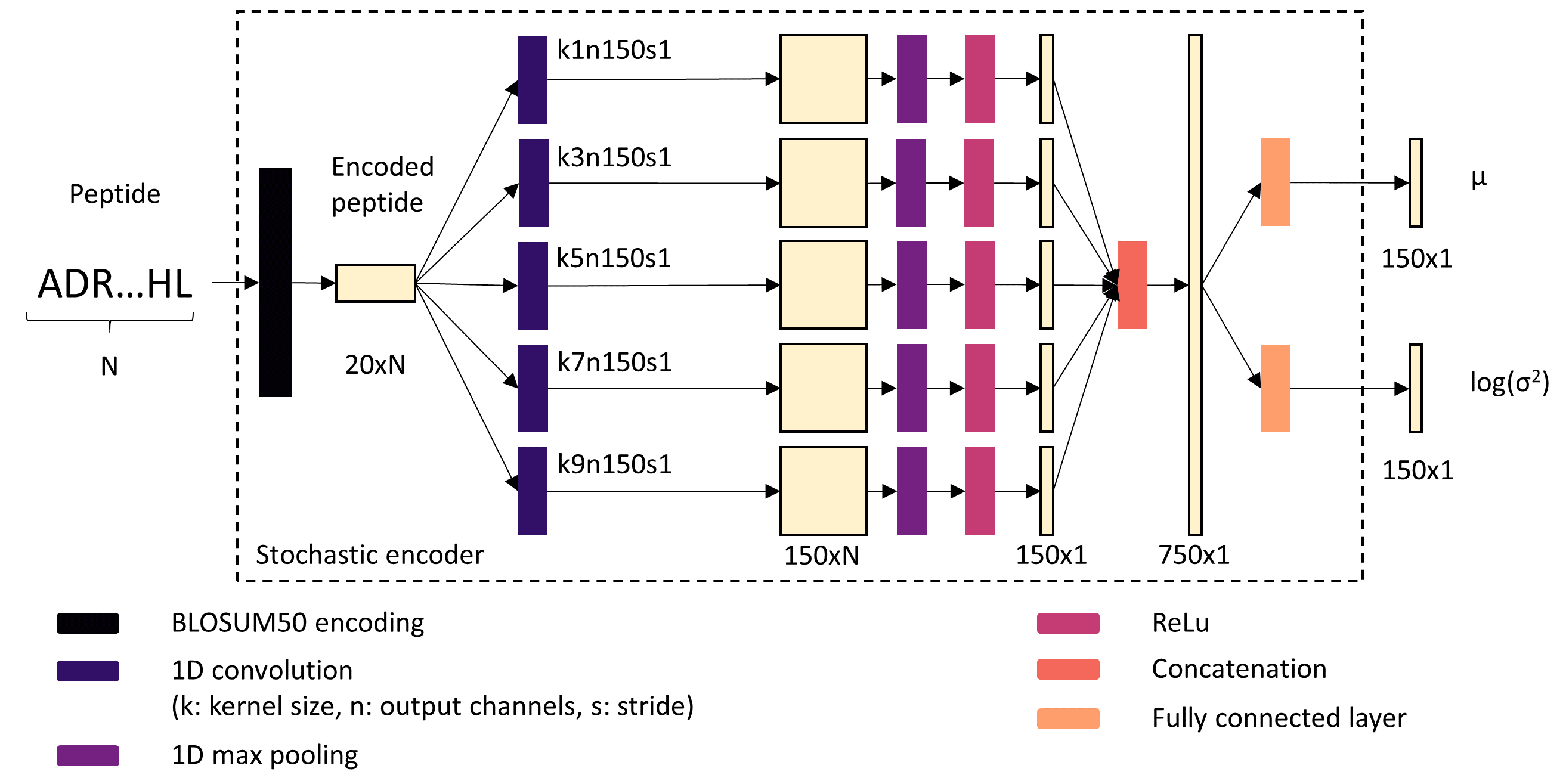

Supplement: btac820_Supplementary_Data [file btac820_supplementary_data.zip › btac820_Supplementary_Data/Sup-Fig-2.png]

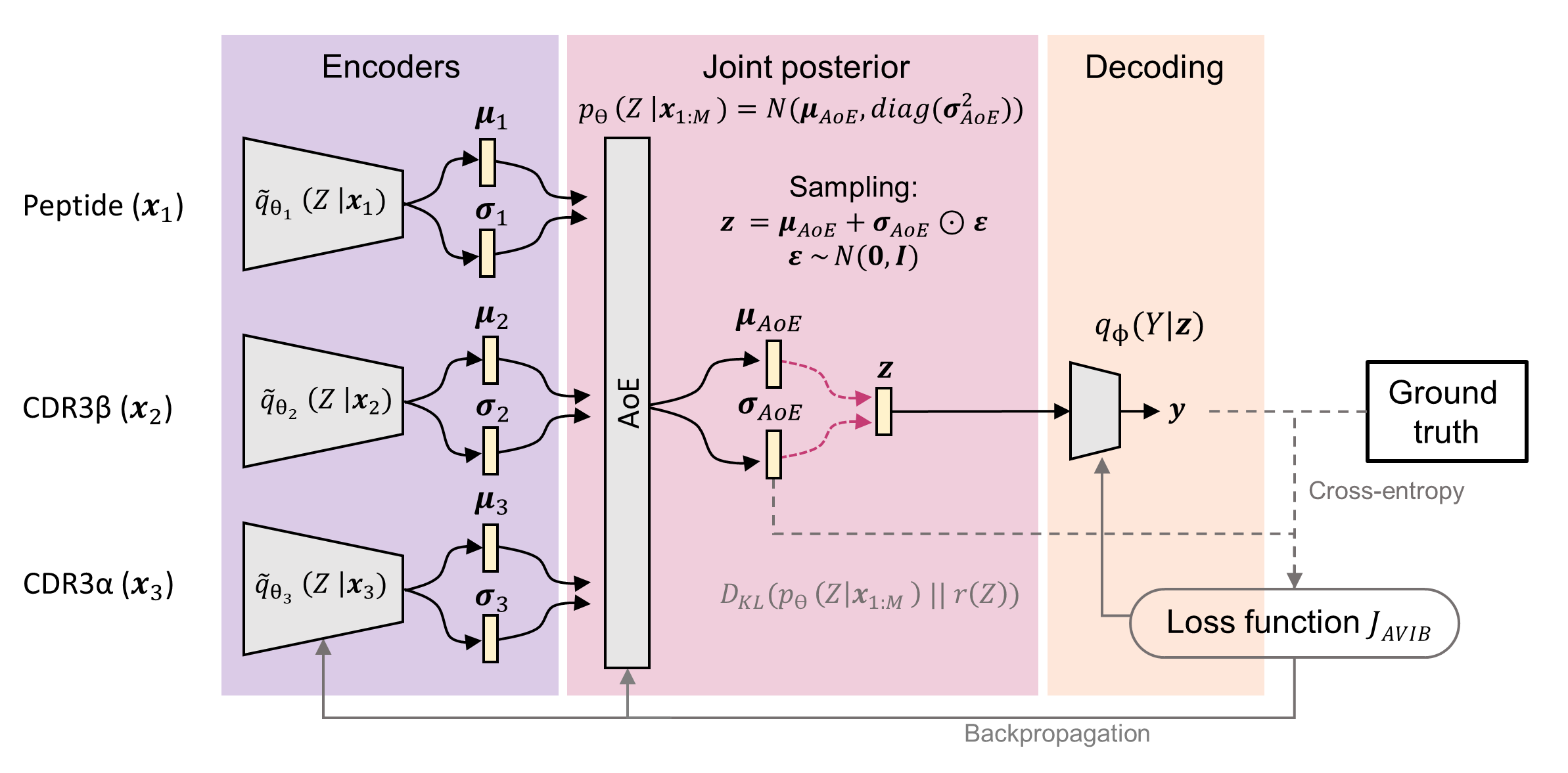

Supplement: btac820_Supplementary_Data [file btac820_supplementary_data.zip › btac820_Supplementary_Data/Sup-Fig-3.png]

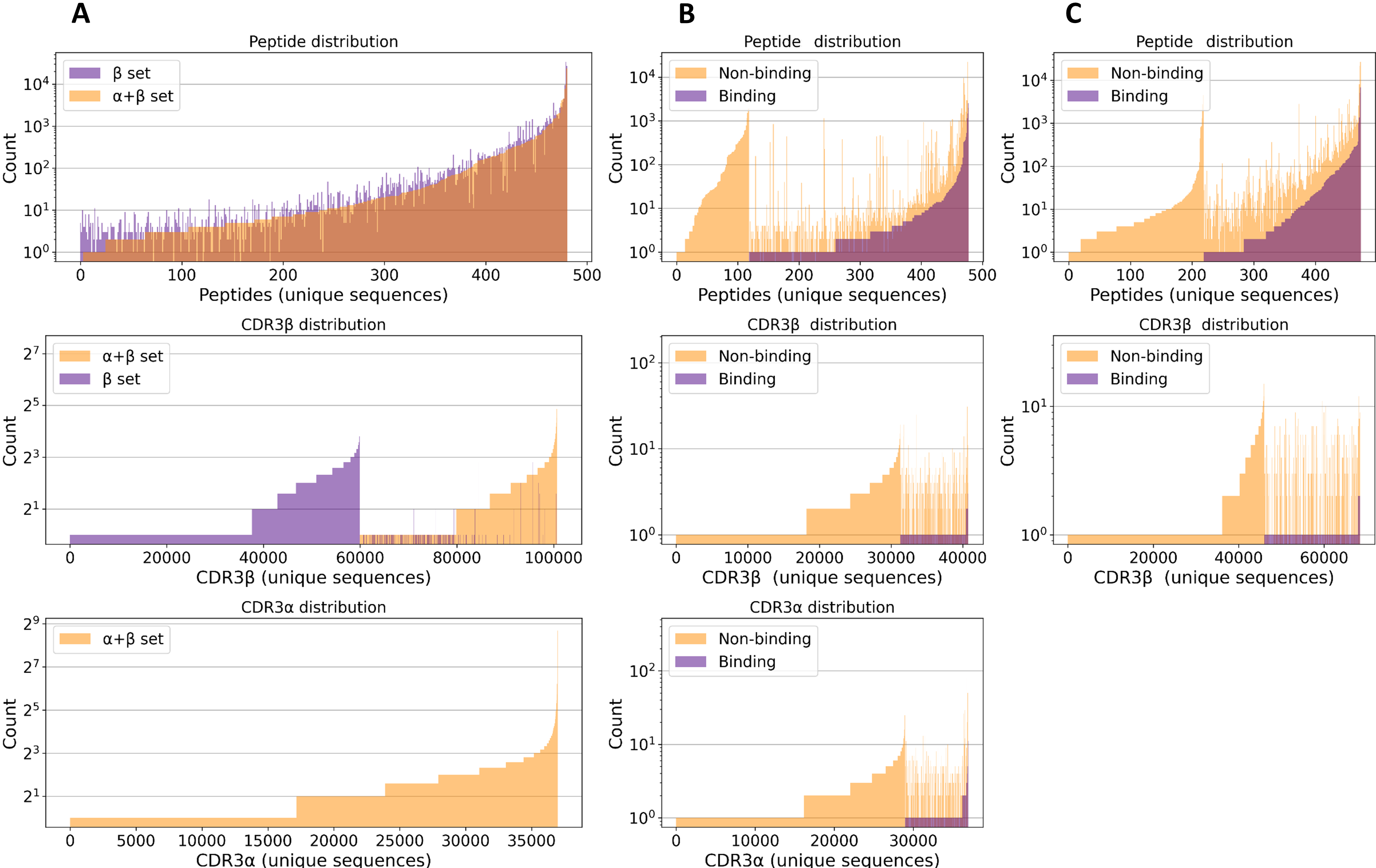

Supplement: btac820_Supplementary_Data [file btac820_supplementary_data.zip › btac820_Supplementary_Data/Sup-Fig-4.png]

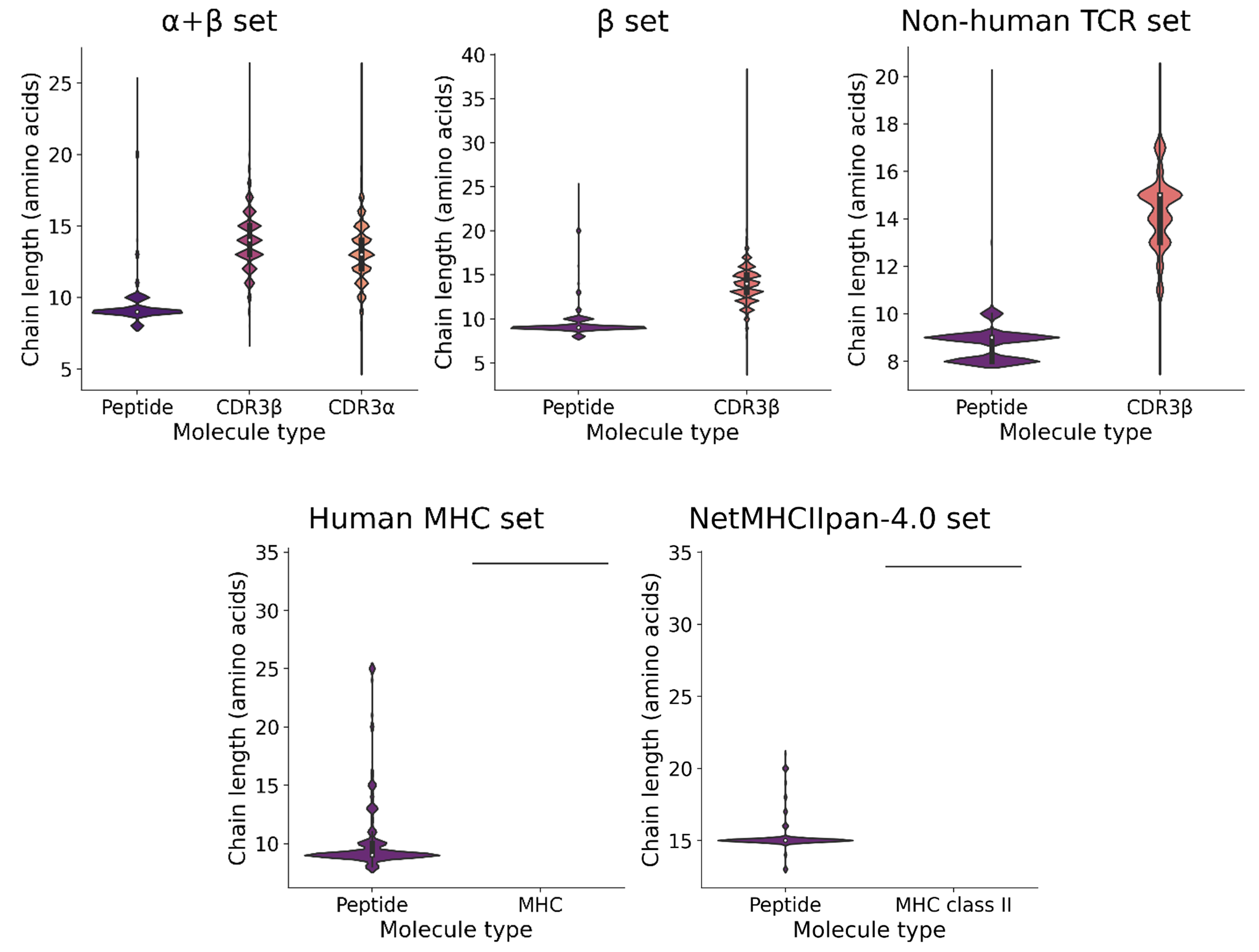

Supplement: btac820_Supplementary_Data [file btac820_supplementary_data.zip › btac820_Supplementary_Data/Sup-Fig-5.png]

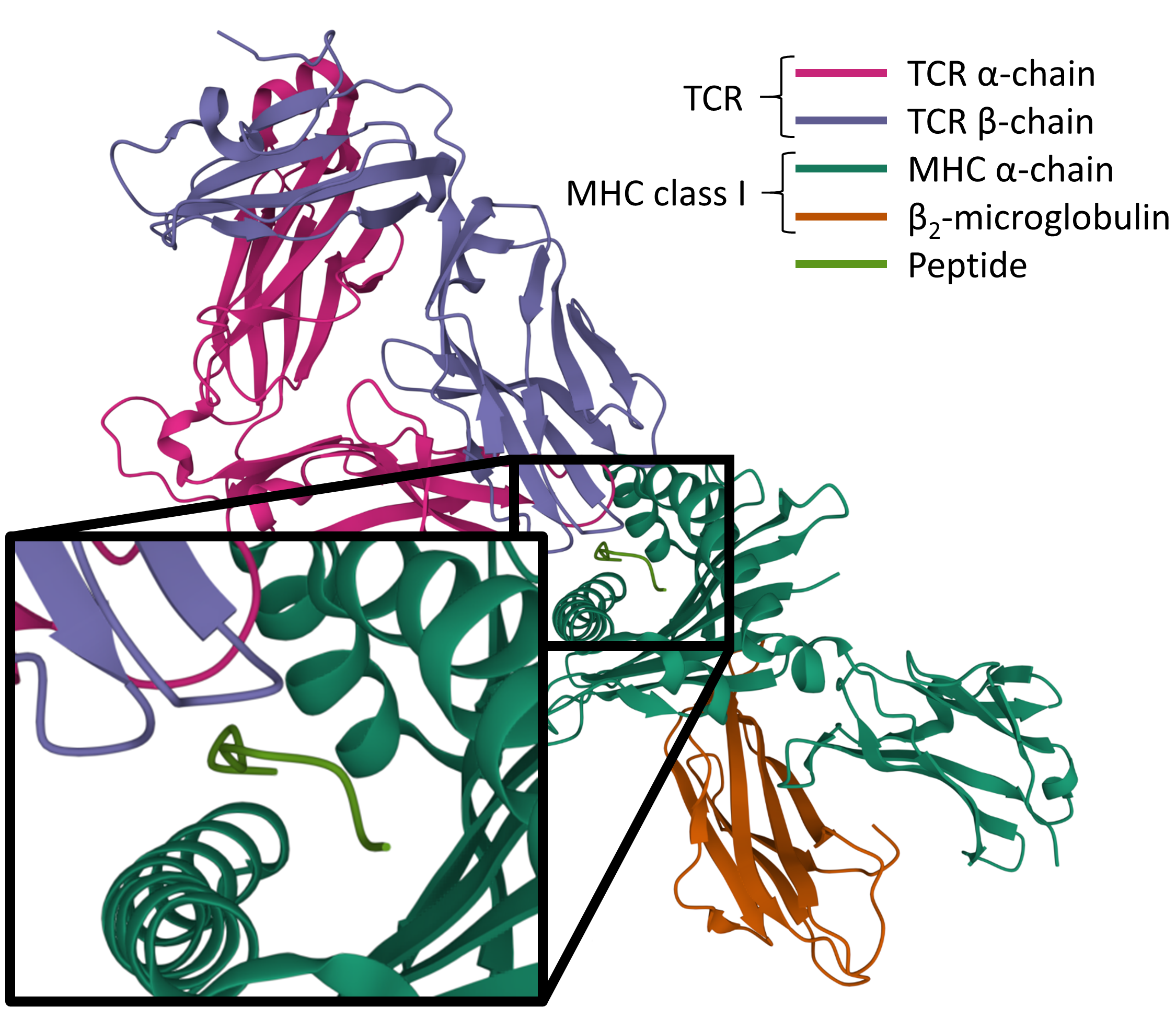

Supplement: btac820_Supplementary_Data [file btac820_supplementary_data.zip › btac820_Supplementary_Data/Supp-Fig-1.png]
